# Immunomodulatory lipid mediator profiling of cerebrospinal fluid following surgery in older adults

**DOI:** 10.1038/s41598-021-82606-5

**Published:** 2021-02-04

**Authors:** Niccolò Terrando, John J. Park, Michael Devinney, Cliburn Chan, Mary Cooter, Pallavi Avasarala, Joseph P. Mathew, Quintin J. Quinones, Krishna Rao Maddipati, Miles Berger, for the MADCO-PC Study Team, Brian Brigman, Jeffrey Browndyke, William M. Bullock, Jessica Carter, Joseph Chapman, Brian Colin, Thomas A. D’Amico, James K. DeOrio, Ramon M. Esclamado, Michael N. Ferrandino, Jeffrey Gadsden, Grant E. Garrigues, Jason Guercio, Ashraf Habib, David H. Harpole, Mathew G. Hartwig, Ehimemen Iboaya, Brant A. Inman, Anver Khan, Sandhya Lagoo-Deenadayalan, Paula S. Lee, Walter T. Lee, John Lemm, Howard Levinson, Christopher Mantyh, David L. McDonagh, John Migaly, Suhail K. Mithani, Eugene Moretti, Judd W. Moul, Mark F. Newman, Brian Ohlendorf, Alexander Perez, Andrew C. Peterson, Glenn M. Preminger, Cary N. Robertson, Sanziana A. Roman, Scott Runyon, Aaron Sandler, Faris M. Sbahi, Randall P. Scheri, S. Kendall Smith, Leonard Talbot, Julie K. M. Thacker, Jake Thomas, Betty C. Tong, Steven N. Vaslef, Nathan Waldron, Xueyuan Wang, Christopher Young

**Affiliations:** 1 Duke University Medical Center, Durham, NC USA; 2 Duke University School of Medicine, Durham, NC USA; 3 Wayne State University, Detroit, MI USA

**Keywords:** Neuroscience, Ageing

## Abstract

Arachidonic acid (AA), docosahexaenoic acid (DHA), and eicosapentaenoic acid (EPA) derived lipids play key roles in initiating and resolving inflammation. Neuro-inflammation is thought to play a causal role in perioperative neurocognitive disorders, yet the role of these lipids in the human central nervous system in such disorders is unclear. Here we used liquid chromatography–mass spectrometry to quantify AA, DHA, and EPA derived lipid levels in non-centrifuged cerebrospinal fluid (CSF), centrifuged CSF pellets, and centrifuged CSF supernatants of older adults obtained before, 24 h and 6 weeks after surgery. GAGE analysis was used to determine AA, DHA and EPA metabolite pathway changes over time. Lipid mediators derived from AA, DHA and EPA were detected in all sample types. Postoperative lipid mediator changes were not significant in non-centrifuged CSF (*p* > 0.05 for all three pathways). The AA metabolite pathway showed significant changes in centrifuged CSF pellets and supernatants from before to 24 h after surgery (*p* = 0.0000247, *p* = 0.0155 respectively), from before to 6 weeks after surgery (*p* = 0.0000497, *p* = 0.0155, respectively), and from 24 h to 6 weeks after surgery (*p* = 0.0000499, *p* = 0.00363, respectively). These findings indicate that AA, DHA, and EPA derived lipids are detectable in human CSF, and the AA metabolite pathway shows postoperative changes in centrifuged CSF pellets and supernatants.

## Introduction

Both animal and human studies show that peripheral tissue trauma induces a significant inflammatory response within the central nervous system^1–4^. Preclinical models suggest that this neuroinflammatory response to surgery may play a causal role in perioperative neurocognitive disorders (PNDs) such as delirium and postoperative cognitive dysfunction/neurocognitive disorder postoperative^5,6^. Indeed, neuroinflammation has detrimental neurocognitive effects in central nervous system (CNS) disorders such as multiple sclerosis, autoimmune encephalitis, and Alzheimer’s disease (AD)^7–9^.

Recent works have also demonstrated that inflammation is actively regulated by polyunsaturated fatty acid-derived lipids known as specialized pro-resolving molecules (SPMs)^10,11^. SPMs, such as lipoxins are derived from arachidonic acid (AA), whereas resolvins, maresins, and protectins, are synthetized from docosahexaenoic acid (DHA) and eicosapentaenoic acid (EPA). These SPM sub-families play different, yet well-orchestrated roles in resolving inflammation, including suppression of NF-kB dependent inflammatory gene expression (reviewed in^12^). SPMs, including aspirin-triggered RvD1 and maresin 1 (MaR1), play key roles in resolving neuroinflammation and reducing memory deficits after surgery in mice^13,14^.

To date, no studies have thoroughly profiled SPMs in the human central nervous system before and after surgery. This represents a necessary first step toward ultimately determining the role of SPMs in humans following surgical recovery, and may have therapeutic implications for conditions like PNDs and other postoperative neurologic complications such as stroke^7,15–17^. To this end, we measured CSF lipid mediators derived from AA, DHA, and EPA before and after surgery in older adults who were enrolled in the prospective cohort study MADCO-PC (Markers of Alzheimer’s Disease and Neurocognitive Outcomes after Perioperative Care)^18^. Further, we performed these measurements on non-centrifuged CSF, centrifuged CSF supernatants, and centrifuged CSF cell pellets to determine the extent to which these mediators are present in the cellular fraction versus aqueous phase of CSF.

## Methods

### Patient enrollment

CSF samples were obtained from patients enrolled in the Markers of Alzheimer’s Disease and neurocognitive Outcomes after Perioperative Care (MADCO-PC) study, which was approved by the Duke IRB and registered on clinicaltrials.gov (NCT01993836) in accordance with the Declaration of Helsinki. MADCO-PC enrolled Duke surgical patients who were English-speaking, ≥ 60 years of age, and scheduled for non-cardiac/non-neurologic surgery under general anesthesia for at least 2 h with a planned postoperative hospital stay of at least 1 night^19^. We excluded patients who were correctional facility inmates, receiving anticoagulation therapy that would preclude lumbar puncture, or had a personal or family history of malignant hyperthermia. Patients who received chemotherapy or who experienced a traumatic brain injury between the baseline and 6-week postoperative study visits were also excluded. Patients who agreed to participate were scheduled for baseline study visits within one month prior to their scheduled surgery and signed informed consent prior to study participation.

### Sample acquisition, processing, and storage

CSF samples were obtained at a baseline study visit ≤ 1 month prior to surgery, and 24 h and 6 weeks after surgery. Lumbar punctures were performed as described^19,20^ using a 25-gauge pencil point spinal needle. A 10-mL polypropylene syringe was used to aspirate CSF from the spinal needle prior to placing the CSF into a 15-mL centrifuge tube (VWR, Radnor, PA, USA; catalog No 10025-686) pre-chilled to 4 °C. Since lipid mediators are largely hydrophobic/lipophilic, we centrifuged CSF samples from a second set of patients from the same study in order to separately measure lipid mediators in the cell pellet vs aqueous supernatant. To accomplish this, CSF samples were centrifuged at 800 g for 10 min at 4 °C. The resulting supernatant was decanted into a separate 15-mL tube that was pre-chilled on ice, and aliquoted into 1.5-mL polypropylene tubes (Sarstedt, VWR; catalog No 10160-142) that were also pre-chilled to 4 °C, and then stored at − 80 °C. For cryopreservation, the resulting cell pellet was re-suspended in 0.5 mL 90% Hyclone heat-inactivated FBS (GE Healthcare Life Sciences, Logan, OR, USA; catalog No. SH30071.03HI) and 10% Hybri-Max DMSO (Sigma, St. Louis, MO, USA; catalog No. D2660-100ML) that was pre-chilled to ~ 4 °C, and placed into a BioCision Cool Cell (Corning, Corning, NY, USA; catalog No. 432000) prior to freezing at − 80 °C. Samples were transferred to long-term storage at − 140 °C after 1 week at − 80 °C.

### LC–MS lipid mediator measurements

SPMs derived from AA, DHA, and EPA were measured using liquid chromatography with mass spectrometry (LC–MS) as outlined^13,21^. For each time point, 3-mL CSF aliquots (non-centrifuged CSF or centrifuged CSF supernatant) or cryopreserved centrifuged CSF pellets were sent to Wayne State University (Detroit, MI, USA) for LC–MS analysis. Samples were assigned anonymous codes; thus, assays were blinded to sample identity. In brief, each sample for each time point was thawed on ice and spiked with internal standards. LC–MS-grade methanol was then added to each sample (15% concentration). Samples were sonicated for 2 min, and then left on ice in the dark for 1 h. Samples were applied to a C18 solid phase extraction cartridge (StrataX C18, 30 mg; Phenomenex, Torrance, CA, USA) that was pre-conditioned with 2 mL methanol and 2 mL water and 15% methanol. Cartridges were washed first with 2 mL 15% methanol in water and then with 2 mL hexane, and dried under vacuum. Cartridges were then eluted with 1 mL methanol with 0.1% formic acid directly into HPLC autosampler vials, and the resulting eluate was evaporated under nitrogen at 25 °C. The resulting residue was reconstituted in methanol, flushed with nitrogen, and stored at − 80 °C.

For LC–MS measurements, samples were thawed at room temperature, and an equal volume of 25 mM aqueous ammonium acetate was added. The mixture was then vortexed and loaded in the autosampler at 15 °C. HPLC was performed on a Prominence XR system (Shimadzu, Kyoto, Japan) using a Luna C18(2), 3 µ, 2.1 × 150 mm column (Phenomenex) with a mobile phase gradient between methanol–water-acetonitrile (10:85:5 v/v) (A) and methanol–water acetonitrile (90:5:5 v/v) (B), each containing 0.1% ammonium acetate. The elution gradient with respect to mobile phase B: 0–1 min 50%, 1–8 min 50–80%, 8–15 min 80–95%, and 15–17 min 95%) and a flow rate of 0.2 mL/min. The HPLC eluate was then introduced to the electrospray ionization (ESI) source of a QTRAP5500 analyzer (SCIEX, Framingham, MA, USA) in negative ion mode, with conditions set at: curtain gas, GS1, and GS2: 35 psi, temperature 600 °C, ion spray voltage − 2500 V, collision gas set at low, declustering potential − 60 V, entrance potential − 7 V. The eluate was monitored by Multiple Reaction Monitoring (MRM) to detect distinct molecular ion-daughter ion combinations. Optimized collisional energies (18–35 eV) and collision cell exit potentials (7–10 V) were used for each MRM transition. Mass spectra were then recorded for each lipid mediator, and the detected peaks were verified via Enhanced Product Ion spectra. Data were collected through Analyst 1.6.2 (SCIEX), and MRM transition chromatograms were quantitated by MultiQuant software (SCIEX) with internal standard signals for each chromatogram used for normalization for recovery and quantitation of each lipid analyte. The resulting area ratios for non-centrifuged CSF, CSF cell pellets, and CSF supernatants were assessed for validity, and retained for analyses only if the signal-to-noise ratio was greater than 3^22^.

### Lipid mediator analysis in non-centrifuged CSF and centrifuged CSF supernatants

For any lipid mediator(s) with a concentration value below the lower limit of detection (i.e., 0.0001 ng/mL), this lower limit of detection (0.0001 ng/mL) was imputed. In non-centrifuged CSF and centrifuged CSF supernatant samples, we considered a lipid analyte to be present only if its median value across patients exceeded the lower limit of detection at one or more timepoints (i.e., pre-operative, 24 h post-operative, or 6 weeks post-operative).

### Lipid mediator analysis in centrifuged CSF pellets

Centrifuged CSF pellets were resuspended in FBS and 10% DMSO (as discussed above) and stored at − 140 °C prior to LC–MS. To address the possibility that FBS with 10% DMSO could contain measurable levels of lipid mediators (which could confound sample measurements), we also measured lipid mediator concentrations in three separate 0.5 mL samples of FBS with 10% DMSO (control diluent; Supplemental Table 1). To ensure that measured lipid mediator values in CSF cell pellets were not due to the addition of the resuspension media (FBS with 10% DMSO), and to account for variance in lipid mediator levels among the 3 samples of FBS with 10% DMSO that we analyzed, we used an additional conservative cutoff threshold. This additional conservative cutoff threshold was set to 10 times the mean of the lipid mediator contained within 0.5 mL FBS with 10% DMSO in nanograms. Thus, lipid mediator values in the centrifuged cell pellet samples were considered present only if they were 10 times higher than the mean quantity of that given lipid in FBS with 10% DMSO and at all time points. Lipid mediator levels in centrifuged CSF cell pellets are reported as the levels originally measured in these samples minus the levels in the cryopreservation media (FBS with 10% DMSO).

### Statistical analysis

Since this was the first study of its kind to systematically measure these lipid mediators in human CSF, there was no a priori data from which to perform a sample size calculation or power analysis. Thus we simply utilized non-centrifuged CSF samples from 92 MADCO-PC study patients (Table 1), and centrifuged CSF pellet samples and supernatants from another 20 MADCO-PC study patients (Table 2).

**Table 1:** Patient characteristics for non-centrifuged samples.

	Non-centrifuged (N = 92)
Age (SD)	68.7 (6.5)
**Race (%)**	
Black or African American	9 (9.8%)
Caucasian/White	82 (89.1%)
Not reported/declined	1 (1.1%)
Sex (Male) (%)	54 (58.7%)
Height (cm) (Q1, Q3)	172.7 [162.8, 180.4]
Weight (kg) (SD)	86.3 (19.7)
BMI (Q1, Q3)	29.1 [24.6, 32.9]
Years of education [Q1, Q3]	16.0 [12.0, 17.8]
**ASA (%)**	
1	1 (1.1%)
2	20 (21.7%)
3	70 (76.1%)
4	1 (1.1%)
**Surgical service (%)**	
Thoracic	10 (10.9%)
General surgery	26 (28.3%)
Gynecology	2 (2.2%)
Orthopedics	18 (19.6%)
Otolaryngology head and neck	2 (2.2%)
Plastic surgery	3 (3.3%)
Urology	31 (33.7%)
**Block Type (%)**	
None	78 (84.8%)
Regional	14 (15.2%)
Epidural	0 (0.0%)
Surgery duration (min) [Q1, Q3]	137.0 [98.0, 193.0]
Case average BIS [Q1, Q3]	45.6 [41.0, 51.7]
Case average aaMAC [Q1, Q3]	0.8 [0.7, 0.8]
Intraoperative propofol dose [Q1, Q3]	320.0 [150.0, 1102.2]
Hospital LOS (Days) [Q1, Q3]	2 [1, 3]

Average bispectral index (BIS) values were calculated from 66 patients in the non-centrifuged group. Case average age-adjusted minimum alveolar concentration (aaMAC) was calculated for those patients who received an inhaled anesthetic for more than half of the case; N = 49 in the non-centrifuged sample group. American Society of Anaesthesiologists scores (ASA) are presented as percentages of non-centrifuged patient samples and centrifuged patient samples. Hospital length of stay (LOS) values are listed in days.

**Table 2:** Patient characteristics for centrifuged samples.

	Centrifuged (N = 20)
Age (SD)	68.2 (5.6)
**Race (%)**	
Black or African American	1 (5.0%)
Caucasian/White	19 (95.0%)
Not reported/declined	0 (0.0%)
Sex (Male) (%)	12 (60.0%)
Height (cm) (Q1, Q3)	172.7 [162.0, 179.1]
Weight (Kg) (SD)	88.3 (20.3)
BMI (Q1, Q3)	28.7 [25.2, 34.5]
Years of education [Q1, Q3]	15.5 [14.0, 16.0]
**ASA (%)**	
1	1 (5.0%)
2	1 (5.0%)
3	18 (90.0%)
4	0 (0.0%)
**Surgical service (%)**	
Thoracic	2 (10.0%)
General surgery	8 (40.0%)
Gynecology	0 (0.0%)
Orthopedics	3 (15.0%)
Otolaryngology head and neck	1 (5.0%)
Plastic surgery	2 (10.0%)
Urology	4 (20.0%)
**Block type (%)**	
None	18 (90.0%)
Regional	2 (10.0%)
Epidural	0 (0.0%)
Surgery duration (min) [Q1, Q3]	135.5 [99.0, 195.0]
Case average BIS [Q1, Q3]	47.2 [42.7, 52.1]
Case average aaMAC [Q1, Q3]	0.8 [0.7, .0.9]
Intraoperative propofol dose [Q1, Q3]	305.0 [150.0, 1469.0]
Hospital LOS (Days) [Q1, Q3]	1 [1, 2]

Average bispectral index (BIS) values were calculated from all 20 patients in the centrifuged samples group. Case average age-adjusted minimum alveolar concentration (aaMAC) was calculated for those patients who received an inhaled anesthetic for more than half of the case; N = 11 patients in the centrifuged samples group. American Society of Anaesthesiologists scores (ASA) are presented as percentages of non-centrifuged patient samples and centrifuged patient samples. Hospital length of stay (LOS) values are listed in days.

LC–MS data for individual lipid mediators in centrifuged CSF pellets and supernatants were analyzed using 2-way repeated measures ANOVAs in R (version 4.0.0). Post hoc tests included testing for factor interactions and Tukey’s Honestly Significant Difference (HSD) test. For individual lipid analytes, family-wise error rate was corrected using the Bonferroni correction.

To study changes in the AA, EPA and DHA derived metabolite pathways over time, LC–MS data were analyzed using Generally Applicable Gene-set Enrichment (GAGE) analysis in the computing language R (version 3.6.2) as outlined^23^ which uses a two-sample *t*-test for pathway significance. Since this analysis was performed to examine changes over time within each lipid (AA, DHA, and EPA) derived metabolite pathway, samples were excluded for this analysis from any patients who did not have samples available at all 3 time points (i.e., preoperative, 24 h postoperative, and 6 weeks postoperative). This resulted in the exclusion of samples from 20 of the 92 patients whose non-centrifuged CSF samples were used for lipid measurements. Values were normalized to have zero mean and unit standard deviation within each group. Multiple comparison corrections were performed for GAGE analysis using the false discovery rate (q-values).

## Results

### Lipid mediator measurements in CSF

Baseline characteristics of the patients whose CSF samples were not centrifuged (N = 92) and those whose CSF samples were centrifuged (yielding CSF cell pellet and CSF supernatant samples; N = 20) are presented in Tables 1 and 2, respectively. Both groups were comprised of older patients (i.e. late 60 s) that were roughly 60% male, and who underwent surgeries over 2 h long under general anesthesia. The two most common types of surgery in both groups were general and urologic surgery.

Non-centrifuged CSF samples had detectable levels of the AA mediators TXB_2_, PGE_2_, 5-HETE, 11-HETE, 12-HETE, 15-HETE, and 5(S),12(S)-DiHETE; the DHA mediators PD1(n-3,DPA), 4-HDoHE, 13-HdoHE, and 14-HdoHE; and the EPA mediators RvE3, 5(S),15(S)-DiHEPE, and 18-HEPE for at least 1 of the observed timepoints (Supplemental Table 2).

For centrifuged CSF cell pellets, the AA mediators PGE_2_, LXA_4_, LTB_4_, 5(S),15(S)-DiHETE, 5(S),12(S)-DiHETE, and 15-epi LXA_4_, the EPA mediator RvE2, and the DHA mediator 10S,17S-DiHDoHE were detectable at all time points (Supplemental Table 3). For centrifuged CSF supernatants, the AA mediators TXB_2_, PGE_2_, PGF_2_α, 5-HETE, 11-HETE, 12-HETE, 15-HETE, 12(S)-HHTrE, and 5(S),12(S)-DiHETE, the EPA mediator 18-HEPE, and the DHA mediators PD1, 4-HDoHE, 13-HDoHE, 14-HDoHE, and 17-HDoHE were detected (i.e. above the lower limit of quantitation) for at least 1 of the observed timepoints (Supplemental Table 4).

### Sample type and timepoint effects on centrifuged cohort lipid mediator levels

**Figure 1:**
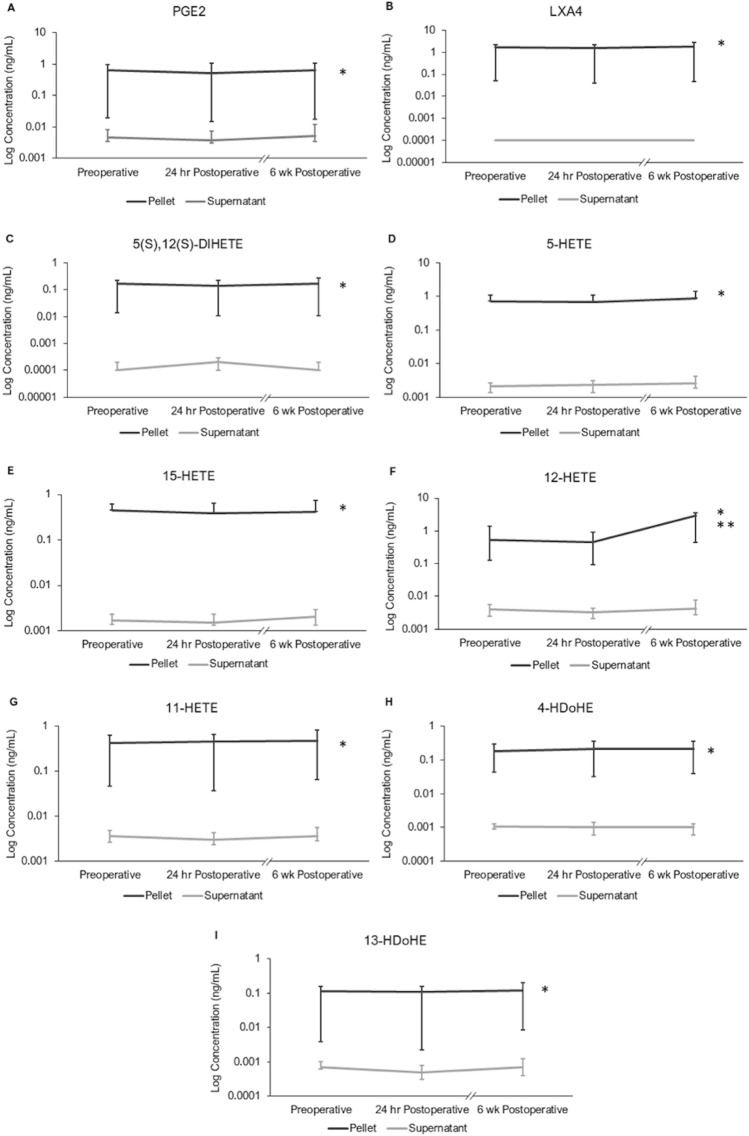
Centrifuged CSF Pellet and Supernatant median log concentrations of lipid mediators over time (N = 20). As supernatant analyte concentrations tended to be lower than pellet analyte concentrations by a few orders of magnitude, a log scale was used to present both sample types. *Indicates a significant (*p* < 0.05) effect of sample type on analyte concentration. **Indicates a significant (*p* < 0.05) effect of both time, and time by sample type interaction for analyte concentration. (**A**) PGE2. (**B**) LXA4. (**C**) 5(S),12(S)-DiHETE. (**D**) 5-HETE. (**E**) 15-HETE. (**F**) 12-HETE. (**G**) 11-HETE. (**H**) 4-HDoHE. (**I**) 13-HDoHE.

ANOVA was performed on lipid mediators whose median analyte concentration values were greater than 0.1 ng/mL in the centrifuged cell pellet and supernatant group: PGE_2_, LXA_4_, 5(S),12(S)-DiHETE, 5-HETE, 15-HETE, 12-HETE, 11-HETE, 4-HDoHE, and 13-HDoHE. Median log concentrations of lipid mediators for the centrifuged samples cohort (i.e. centrifuged CSF pellets and supernatants) were plotted over each timepoint (Fig. 1). Sample type (i.e. centrifuged CSF pellet vs centrifuged CSF supernatant) had a significant effect on lipid mediator concentrations for all analytes (*p* > 0.05). Timepoint (pre-op, 24 h post-op, and 6 weeks post-op) did not have a significant effect on lipid mediator concentration except for 12-HETE. Post-hoc testing for 12-HETE showed a significant increase in its concentration from 24 h post-op to 6 weeks post-op (*p* = 0.0012). There was also a ~ 180% increase in 12-HETE level from before to 6 weeks after surgery (*p* = 0.0014), though there were no significant differences in 12-HETE levels between the pre-op and 24 h post-op timepoints (*p* > 0.05). Interaction between sample type and timepoint was not significant for any analyte (*p* > 0.05), except for 12-HETE at 6 weeks post-op (*p* = 1.10E^−8^).

### GAGE analysis for pathway changes

**Figure 2:**
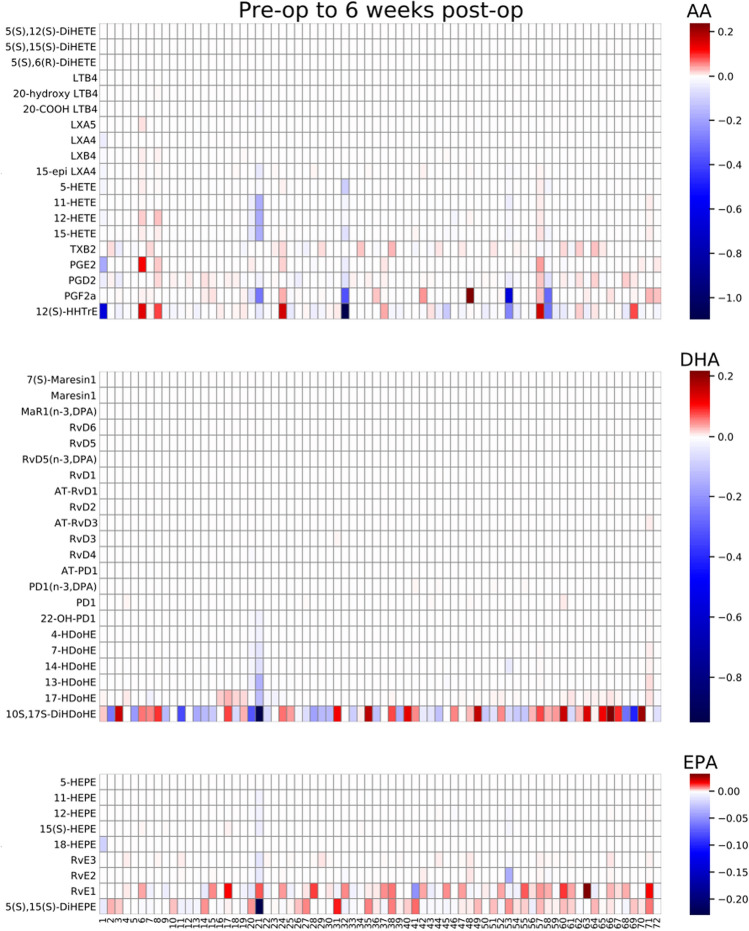
Non-centrifuged CSF heatmaps for AA, DHA, and EPA derived metabolite pathway changes from pre-op to 6 weeks post-op (N = 72). Patients are listed as numbers on the x-axis for each heatmap. Heatmap scale numbers represent natural log ratios of analyte concentrations from pre-op to 6 weeks post-op.

**Figure 3:**
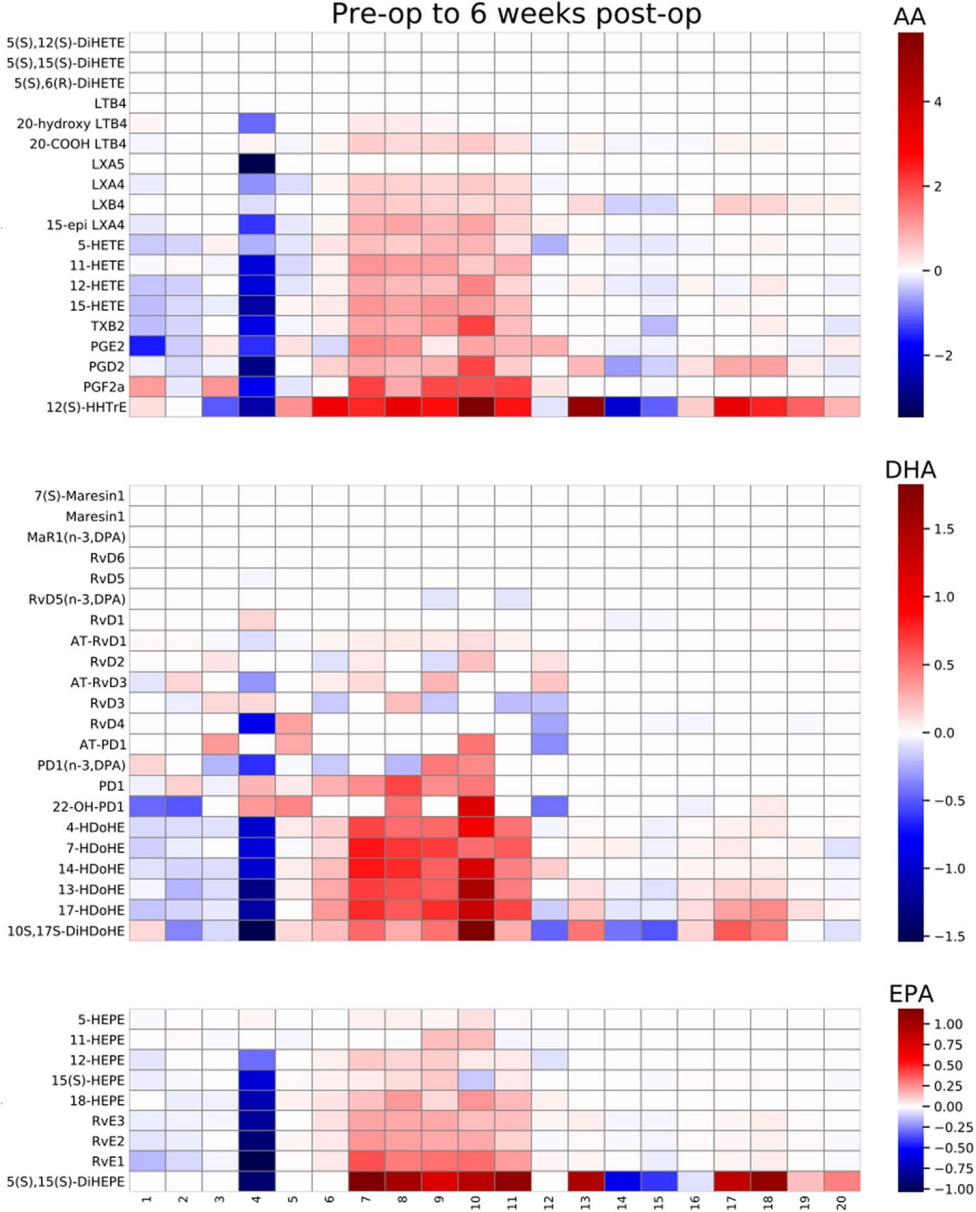
Centrifuged CSF cell pellet heatmaps for AA, DHA, and EPA derived metabolite pathway changes from pre-op to 6 weeks post-op (N = 20). Patients are listed as numbers on the x-axis for each heatmap. Heatmap scale numbers represent natural log ratios of analyte concentrations from pre-op to 6 weeks post-op.

**Figure 4:**
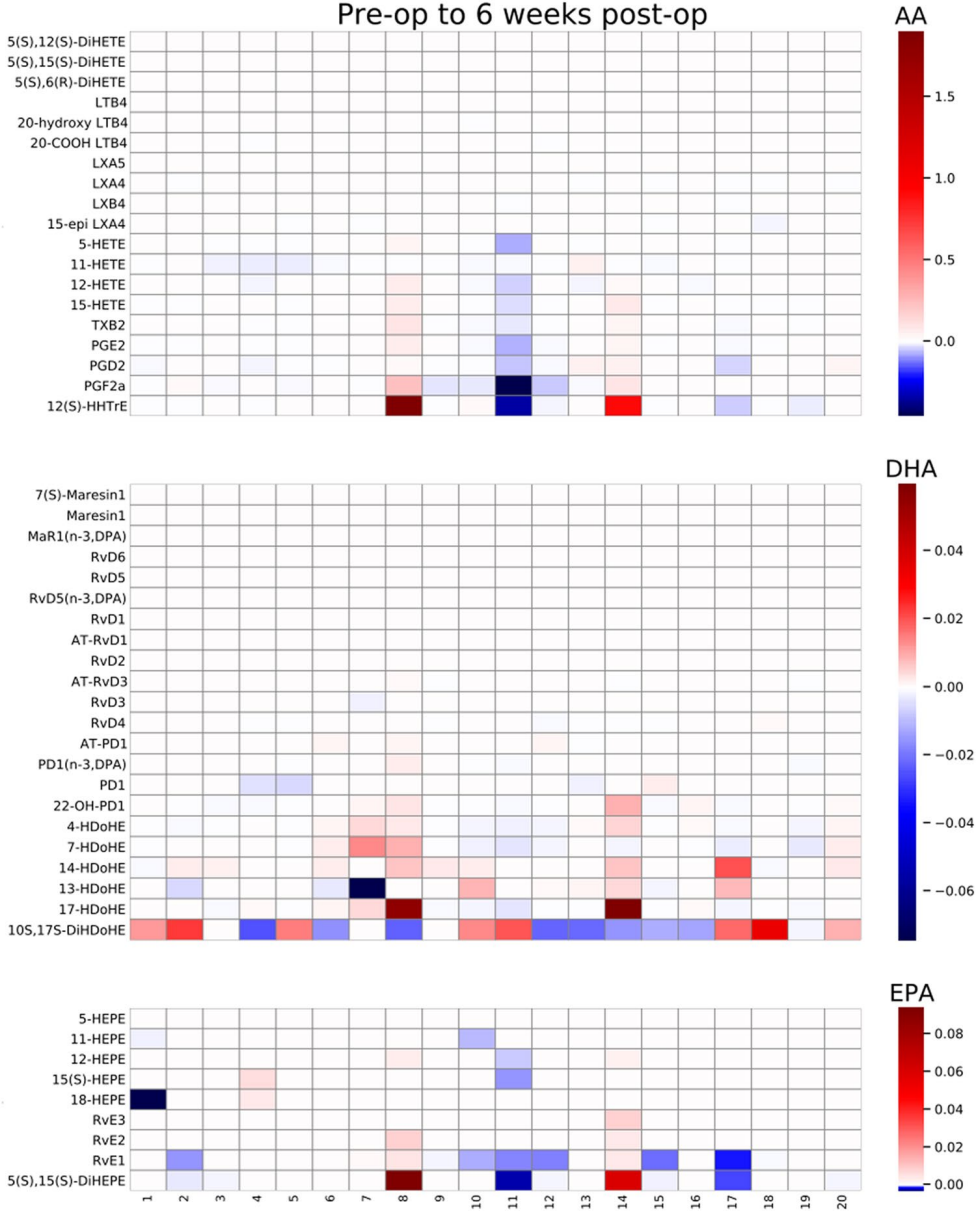
Centrifuged CSF supernatant heatmaps for AA, DHA, and EPA derived metabolite pathway changes from pre-op to 6 weeks post-op (N = 20). Patients are listed as numbers on the x-axis for each heatmap. Heatmap scale numbers represent natural log ratios of analyte concentrations from pre-op to 6 weeks post-op.

GAGE analysis was performed to measure changes within the metabolite pathway of each fatty acid (AA, EPA, and DHA), in each sample type (non-centrifuged CSF, centrifuged CSF cell pellets, centrifuged CSF supernatants) over each time period. These time-period comparisons were made from before to 24 h after surgery, before to 6 weeks after surgery, and 24 h after surgery to 6 weeks after surgery (see Table 3 for *p* and q-values). In both centrifuged CSF cell pellets and supernatants, significant changes were seen in the AA derived metabolite pathway over all 3 time periods (q < 0.05 for each). In contrast, the DHA and EPA derived metabolite pathways did not show changes over any of the time periods in centrifuged CSF cell pellets or supernatants. Non-centrifuged CSF showed no significant changes in any of the 3 pathways. AA, DHA and EPA derived metabolite changes for non-centrifuged CSF (Fig. 2) and centrifuged CSF cell pellets (Fig. 3) and supernatants (Fig. 4) from pre-op to 6 weeks post-op are presented as heatmaps. Additional heatmaps comparing AA, EPA and DHA derived metabolite pathway changes from pre-op to 24 h post-op and 24 h post-op to 6 weeks post-op are presented as supplemental figures for non-centrifuged CSF (Supplemental Fig. 1), centrifuged CSF pellets (Supplemental Fig. 2) and supernatants (Supplemental Fig. 3).

**Table 3:** GAGE analysis for AA, DHA, and EPA derived metabolite pathway changes over the indicated time periods.

Lipid derived pathway	Time period	Non-centrifuged CSF		CSF cell pellet		CSF supernatant	
		*p*-value	q-value	*p*-value	q-value	*p*-value	q-value
AA	Before surgery to 24 h after surgery	0.373	0.851	0.0000247	0.0000741	0.0155	0.0464
	Before surgery to 6 weeks after surgery	0.497	0.778	0.0000497	0.000149	0.00239	0.00716
	24 h after surgery to 6 weeks after surgery	0.200	0.601	0.0000499	0.000150	0.00363	0.0109
DHA	Before surgery to 24 h after surgery	0.567	0.567	1.00	1.00	0.922	1.00
	Before surgery to 6 weeks after surgery	0.519	0.778	1.00	1.00	0.973	1.00
	24 h after surgery to 6 weeks after surgery	0.723	1.00	1.00	1.00	0.978	1.00
EPA	Before surgery to 24 h after surgery	1.00	1.00	1.00	1.00	1.00	1.00
	Before surgery to 6 weeks after surgery	1.00	1.00	1.00	1.00	1.00	1.00
	24 h after surgery to 6 weeks after surgery	1.00	1.00	0.999	1.00	1.00	1.00

## Discussion

Here, we provide evidence that AA-derived pathway mediators showed dynamic changes from before to after surgery in centrifuged CSF supernatants and in cell pellets. Further, we found that SPMs derived from the EPA, DHA, and AA pathways are detectable in centrifuged CSF supernatants, centrifuged CSF cell pellets, and non-centrifuged CSF samples obtained from older adults before and after surgery. A mix of pro- and anti-inflammatory lipid mediators was detected in each sample type (e.g., PGE_2_ and 10S,17S-DiHDoHE in CSF cell pellets) across all time points, consistent with the notion that inflammation is a delicate balance between inflammatory (pro/anti) and pro-resolving factors^11^. Further, we provide the first evidence that human CSF displays dynamic postoperative lipid mediator changes in the AA pathway, but not in the EPA or DHA pathways.

To our knowledge, this is the first comparison of lipid mediator levels in centrifuged CSF cell pellets, supernatants, and non-centrifuged CSF samples. Notably, lipid mediators observed in centrifuged CSF cell pellets were higher than those seen in centrifuged CSF supernatants, even after subtraction of baseline lipid mediator levels in FBS + 10% DMSO. This suggests that SPMs are likely present at higher concentrations in the CSF cellular/pellet fraction than in aqueous CSF. This finding is analogous to the results of prior studies that measured plasma SPM levels; while SPMs can be generated and act in situ in blood (reviewed in^12^), SPMs tend to be generated at higher concentrations locally at sites of active inflammation, and become diluted when they enter circulation resulting in lower SPM concentrations in plasma^24^. The observation that centrifuged CSF pellets showed enrichment of lipid mediators also suggests a compartmentalization of these molecules within cells as opposed to the surrounding fluid. That the enzymes, such as 5-LO (reviewed in^25^) and 15-LOX-1^26^, responsible for the endogenous production of SPMs are present within leukocytes lends further credence to this finding. Indeed, macrophages have been shown to switch from pro-inflammatory molecule generation to SPM production via the action of HMGB1-C1q^27^; since this happens via conversion of polyunsaturated fatty acids, the substrates for this enzymatic action must be available within cells.

While prior studies have measured multiple individual lipid mediator in CSF such as RvD1 and LTB4^7,16^, here we assayed 50 analytes derived from AA, DHA and EPA at multiple timepoints, which allowed for detailed metabolite pathway analysis. As a result, we were able to identify significant changes in the AA pathway in patients after surgery. Additionally, by using LC–MS we improved the detection sensitivity and specificity for these lipid mediators, as compared to ELISA-based assays that may lack molecular specificity due to antibody cross-reactivity^28,29^.

Here we have described postoperative changes in the AA pathway, but not in the EPA or DHA pathways, in human CSF after non-cardiac/non-neurologic surgery. Indeed, the AA pathway is involved in the generation of many pro-inflammatory lipid mediators including leukotrienes and prostaglandins^30^. However, it also promotes resolution by triggering mediators such as lipoxins, which are implicated in the resolving inflammation and are dysregulated in murine AD models^31^. In fact, levels of AA-derived SPMs such as LXA_4_ are also reduced in patients with AD, and lower CSF levels of LXA_4_ and RvD1 correlate with mini-mental status exam scores as well as postmortem expression in the hippocampus^15^.

Neuroinflammation is a key hallmark of AD and also occurs following peripheral trauma, such as anesthesia and surgery^4,13,32^. Changes in select lipid mediators have been described following surgery in humans. For example, upregulation of AA-derived prostaglandins has been noted in the aqueous humor of patients undergoing cataract surgery^33^. Prior studies have also found decreased plasma levels of SPMs, including the AA-derived LXA_4_ and D-series resolvins, 24 h after hepatobiliary surgery^34^. In mouse models, treatment with exogenous pro-resolving lipid mediators attenuated microglial activation and improved hippocampal-dependent memory function following orthopedic surgery^13,14^. These observations have led to the hypothesis that neuroinflammation plays a key etiologic role in human PNDs, which often complicate the recovery of older adults. Whether detecting and potentially modulating specific lipid biomarkers after surgery in the human CNS would predict or even treat PNDs warrants further investigation.

Indeed, a detailed characterization of SPMs from the AA, EPA, and DHA pathways in human CSF may contribute to novel biomarkers not only relevant to PNDs but other neurocognitive disorders, especially given the increasing evidence for inflammatory dysregulation in various neurologic disease states^35,36^. Post-mortem examination of brains from patients with AD demonstrated increased expression of the LTB_4_ and RvE1 receptor BLT1^35^ in the basal forebrain, entorhinal cortex, and hippocampus, which are all brain regions that show neuropathology changes in AD. This increase in BLT1 expression has been interpreted as evidence for a disrupted inflammatory resolution pathway in AD. Likewise, increased CSF 15-HETE and PGE_2_ levels (two pro-inflammatory AA-derived mediators) have been found in patients with active multiple sclerosis^36^. Clearly, more studies will be necessary to understand the biology, timing, and function of immunomodulatory lipid level changes in the human CNS in multiple neurocognitive disease states. Our data from 50 lipid mediators detected by LC–MS demonstrate the feasibility of this approach for such studies.

This study has at least four limitations. First, the sample size for centrifuged CSF samples was relatively low (N = 20), although the non-centrifuged CSF sample size (N = 90) was similar in size to prior SPM CSF investigations using LC–MS^16^. Second, the non-centrifuged CSF samples and centrifuged CSF samples were obtained from two different patient cohorts. The 10–12-mL volume of CSF obtained per patient in both of these cohorts is larger than the CSF volume obtained in many other studies^37–39^. Yet, this 10–12-mL volume is too small to split into 2 parts, one for centrifugation and one without centrifugation, since it takes a full 10–12 mL of CSF to generate a sufficient cell pellet after centrifugation^19^. Thus, it would be difficult if not impossible to measure lipid mediators in individual human CSF samples split so that half could be processed with centrifugation, and half without centrifugation. Patient and/or procedural differences between these two cohorts may explain why AA pathway changes were seen in centrifuged CSF pellet and supernatant samples but not in non-centrifuged CSF. Alternatively, since the highest concentrations of these lipids were detected in the cell pellets, the levels in non-centrifuged CSF samples may simply be too dilute to measure biologically active intracellular changes in these lipid levels. Third, the CSF samples studied here were from patients enrolled in the MADCO-PC study^18^; thus, these data cannot necessarily be generalized beyond this population (i.e., patients age 60 and over undergoing major non-cardiac/non-neurologic surgery). Fourth, it is unclear whether the changes in CNS lipid mediator levels observed here are driven by peripheral^40^ factors that cross the blood–brain barrier, or by factors that originate within the CNS itself^15^, or both. This is a general question that has been posed about the origin of neuroinflammation across multiple neurologic disease states, and applies not only to the immunomodulatory lipid pathways studied here but also to more “traditional” neuroinflammatory mediators such as cytokines that have been more extensively studied^41^.

In conclusion, we have reported dynamic perioperative changes in the CSF lipidome of older surgery patients. The levels of these mediators and the variance among them provides essential preliminary data for powering future studies to decipher their role in various disease states. Such future studies will be required to clarify the role of these mediators in PNDs, and their therapeutic potential for curtailing neuroinflammation and improving cognition in neurologic conditions.

## Supplementary Information

Supplementary Information 1.

## Data Availability

All datasets are available in the supplementary material section. All methods were carried out in accordance with relevant guidelines and regulations for the present study. All experimental protocols were approved by Duke IRB.All datasets are available in the supplementary material section. All methods were carried out in accordance with relevant guidelines and regulations for the present study. All experimental protocols were approved by Duke IRB.

## Abbreviations

10S,17S-DiHDoHE: (4*Z*,7*Z*,10*S*,11*E*,13*Z*,15*E*,17*S*,19*Z*)-10,17-Dihydroxydocosa-4,7,11,13,15,19-hexaenoic acid
11-HEPE: (5*Z*,8*Z*,12*E*,14*Z*,17*Z*)-11-Hydroxyicosa-5,8,12,14,17-pentaenoic acid
11-HETE: (5*Z*,8*Z*,12*E*,14*Z*)-11-Hydroxyicosa-5,8,12,14-tetraenoic acid
12(S)-HHTrE: (5*Z*,8*E*,10*E*,12*S*)-12-Hydroxyheptadeca-5,8,10-trienoic acid
12-HEPE: (5*Z*,8*Z*,10*E*,14*Z*,17*Z*)-12-Hydroxyicosa-5,8,10,14,17-pentaenoic acid
12-HETE: (5*E*,8*E*,10*E*,14*E*)-12-Hydroxyicosa-5,8,10,14-tetraenoic acid
13-HDoHE: (4*Z*,7*Z*,10*Z*,14*E*,16*Z*,19*Z*)-13-Hydroxydocosa-4,7,10,14,16,19-hexaenoic acid
14-HDoHE: (4*Z*,7*Z*,10*Z*,12*E*,16*Z*,19*Z*)-14-Hydroxydocosa-4,7,10,12,16,19-hexaenoic acid
15(S)-HEPE: (5*Z*,8*Z*,11*Z*,13*E*,15*S*,17*Z*)-15-Hydroxyicosa-5,8,11,13,17-pentaenoic acid
15-epi LXA4: (5*S*,6*R*,7*E*,9*E*,11*Z*,13*E*,15*R*)-5,6,15-Trihydroxyicosa-7,9,11,13-tetraenoic acid
15-HETE: (5*Z*,8*Z*,11*Z*,13*E*)-15-Hydroxyicosa-5,8,11,13-tetraenoic acid
17-HDoHE: (4*Z*,7*Z*,10*Z*,13*Z*,15*E*,19*Z*)-17-Hydroxydocosa-4,7,10,13,15,19-hexaenoic acid
18-HEPE: (5*Z*,8*Z*,11*Z*,14*Z*,16*E*)-18-Hydroxyicosa-5,8,11,14,16-pentaenoic acid
20-COOH LTB4: (5*R*,6*E*,8*Z*,10*E*,12*S*,14*Z*)-5,12-Dihydroxyhenicosa-6,8,10,14-tetraenedioic acid
20-hydroxy LTB4: (5*S*,6*Z*,8*E*,10*E*,12*R*,14*Z*)-5,12,20-Trihydroxyicosa-6,8,10,14-tetraenoic acid
22-OH-PD1: (4*Z*,7*Z*,10*R*,11*E*,13*E*,15*Z*,17*S*,19*Z*)-10,17,22-Trihydroxydocosa-4,7,11,13,15,19-hexaenoic acid
4-HDoHE: (5*E*,7*Z*,10*Z*,13*Z*,16*Z*,19*Z*)-4-Hydroxydocosa-5,7,10,13,16,19-hexaenoic acid
5(S),6(R)-DiHETE: (5*S*,6*R*,7*E*,9*E*,11*Z*,14*Z*)-5,6-Dihydroxyicosa-7,9,11,14-tetraenoic acid
5(S),12(S)-DiHETE: (5*S*,6*E*,8*E*,10*E*,12*S*,14*Z*)-5,12-Dihydroxyicosa-6,8,10,14-tetraenoic acid
5(S),15(S)-DiHEPE: 5S,15S-dihydroxyeicosapentaenoic acid
5(S),15(S)-DiHETE: (5*S*,6*E*,8*Z*,11*Z*,13*E*,15*S*)-5,15-Dihydroxyicosa-6,8,11,13-tetraenoic acid
5-HEPE: (6*E*,8*Z*,11*Z*,14*Z*,17*Z*)-5-Hydroxyicosa-6,8,11,14,17-pentaenoic acid
5-HETE: (5*S*,6*E*,8*Z*,11*Z*,14*Z*)-5-Hydroxyicosa-6,8,11,14-tetraenoic acid
7(S)-Maresin1: (4*Z*,7*S*,8*E*,10*E*,12*Z*,14*S*,16*Z*,19*Z*)-7,14-Dihydroxydocosa-4,8,10,12,16,19-hexaenoic acid
7-HDoHE: (4*Z*,8*E*,10*Z*,13*Z*,16*Z*,19*Z*)-7-Hydroxydocosa-4,8,10,13,16,19-hexaenoic acid
AA: Arachidonic acid
AD: Alzheimer’s Disease
AT-PD1: Aspirin triggered-protectin D1
AT-RvD1: Aspirin triggered-resolvin D1
AT-RvD3: Aspirin triggered-resolvin D3
CNS: Central nervous system
CSF: Cerebrospinal fluid
DHA: Docosahexaenoic acid
DMSO: Dimethyl sulfoxide
EPA: Eicosapentaenoic acid
FBS: Fetal bovine serum
GAGE: Generally applicable gene-set enrichment
LC–MS: Liquid chromatography mass spectrometry
LTB4: Leukotriene B4
LXA4: Lipoxin A4
LXA5: Lipoxin A5
LXB4: Lipoxin B4
MaR1: Maresin 1
MaR1(n-3,DPA): N-3 docosapentaenoic acid derived maresin 1
PD1: Protectin D1
PD1(n-3,DPA): N-3 docosapentaenoic acid derived protectin D1
PGD2: Prostaglandin D2
PGE2: Prostaglandin E2
PGF2a: Prostaglandin F2alpha
PNDs: Perioperative neurocognitive disorders
RvD1: Resolvin D1
RvD2: Resolvin D2
RvD3: Resolvin D3
RvD4: Resolvin D4
RvD5: Resolvin D5
RvD5(n-3,DPA): N-3 docosapentaenoic acid derived resolvin D5
RvD6: Resolvin D6
RvE1: Resolvin E1
RvE2: Resolvin E2
RvE3: Resolvin E3
SPM: Specialized pro-resolving molecules
TXB2: Thromboxane B2
