# Supplementary material for: Immunomodulatory lipid mediator profiling of cerebrospinal fluid following surgery in older adults
Source: Sci Rep. 2021 Feb 4;11:3047. doi: 10.1038/s41598-021-82606-5 (PMC7862598; doi:10.1038/s41598-021-82606-5)

**Immunomodulatory Lipid Mediator Profiling of Cerebrospinal Fluid Following Surgery in Older Adults**

**Niccolò Terrando^1,^^, John J. Park^2,^^, Michael Devinney^1^, Cliburn Chan^1^, Mary Cooter^1^, Pallavi Avasarala^1^, Joseph P. Mathew^1^, Quintin J. Quinones^1^, Krishna Rao Maddipati^3^, and Miles Berger^1,*^ for the MADCO-PC Study Team**

^1^Duke University Medical Center, Durham, NC, USA

^2^Duke University School of Medicine, Durham, NC, USA

^3^Wayne State University, Detroit, MI, USA

^These authors contributed equally to this work.

^*^Corresponding author

miles.berger@duke.edu

**Supplemental Section**

Supplemental Table 1: Baseline lipid mediator levels in 0.5 ml samples of FBS + 10% DMSO using LC-MS.

| **Metabolome** | **Lipid** | **FBS + 10% DMSO** | | | **Mean (ng/ml)** | **Standard Deviation (ng/ml)** |
| --- | --- | --- | --- | --- | --- | --- |
|  |  | **Sample 1 (ng/ml)** | **Sample 2 (ng/ml)** | **Sample 3 (ng/ml)** |  |  |
| **AA** | **TXB2** | 41.94061 | 45.28878 | 45.87063 | 44.36667 | 2.12108 |
|  | **PGF2a** | 0.32380 | 0.36859 | 0.34753 | 0.34664 | 0.02241 |
|  | **PGE2** | 0.0001 | 0.0001 | 0.0001 | 0.0001 | 0.00000 |
|  | **PGD2** | 0.0001 | 0.0001 | 0.0001 | 0.0001 | 0.00000 |
|  | **LXB4** | 0.0001 | 0.0001 | 0.0001 | 0.0001 | 0.00000 |
|  | **LXA5** | 0.0001 | 0.0001 | 0.0001 | 0.0001 | 0.00000 |
|  | **LXA4** | 0.0001 | 0.0001 | 0.0001 | 0.0001 | 0.00000 |
|  | **LTB4** | 0.16100 | 0.12306 | 0.09979 | 0.12795 | 0.03089 |
|  | **5(S),6(R)-DiHETE** | 0.06204 | 0.04664 | 0.0001 | 0.03624 | 0.03229 |
|  | **5(S),15(S)-DiHETE** | 0.0001 | 0.0001 | 0.02664 | 0.00890 | 0.01536 |
|  | **5(S),15(S)-DiHEPE** | 0.20950 | 0.18959 | 0.17542 | 0.19151 | 0.01712 |
|  | **5(S),12(S)-DiHETE** | 0.32203 | 0.26264 | 0.26160 | 0.28209 | 0.03459 |
|  | **5-HETE** | 3.69072 | 4.24013 | 3.65718 | 3.86268 | 0.32731 |
|  | **20-hydroxy LTB4** | 0.0001 | 0.0001 | 0.0001 | 0.0001 | 0.00000 |
|  | **20-COOH LTB4** | 0.0001 | 0.0001 | 0.0001 | 0.0001 | 0.00000 |
|  | **15-HETE** | 3.15171 | 3.14304 | 3.12226 | 3.13900 | 0.01514 |
|  | **15-epi LXA4** | 0.0001 | 0.0001 | 0.0001 | 0.0001 | 0.00000 |
|  | **12(S)-HHTrE** | 1.62139 | 1.60464 | 1.48168 | 1.56924 | 0.07629 |
|  | **12-HETE** | 44.27310 | 51.58800 | 48.37466 | 48.07859 | 3.66643 |
|  | **11-HETE** | 3.08955 | 3.26215 | 2.92451 | 3.09207 | 0.16883 |
| **EPA** | **RvE3** | 0.03450 | 0.0001 | 0.0001 | 0.01152 | 0.01990 |
|  | **RvE2** | 0.0001 | 0.0001 | 0.0001 | 0.0001 | 0.00000 |
|  | **RvE1** | 0.0001 | 0.0001 | 0.0001 | 0.0001 | 0.00000 |
|  | **5-HEPE** | 0.11033 | 0.10443 | 0.09351 | 0.10276 | 0.00854 |
|  | **18-HEPE** | 0.06424 | 0.06644 | 0.05846 | 0.06305 | 0.00412 |
|  | **15(S)-HEPE** | 0.09266 | 0.09227 | 0.08225 | 0.08906 | 0.00590 |
|  | **12-HEPE** | 2.18705 | 2.53192 | 2.44072 | 2.38656 | 0.17870 |
|  | **11-HEPE** | 0.06850 | 0.06604 | 0.06417 | 0.06624 | 0.00217 |
| **DHA** | **RvD6** | 0.0001 | 0.0001 | 0.0001 | 0.0001 | 0.00000 |
|  | **RvD5(n-3,DPA)** | 0.02170 | 0.01576 | 0.01448 | 0.01732 | 0.00385 |
|  | **RvD5** | 0.0001 | 0.0001 | 0.0001 | 0.0001 | 0.00000 |
|  | **RvD4** | 0.0001 | 0.0001 | 0.0001 | 0.0001 | 0.00000 |
|  | **RvD3** | 0.0001 | 0.0001 | 0.0001 | 0.0001 | 0.00000 |
|  | **RvD2** | 0.0001 | 0.0001 | 0.0001 | 0.0001 | 0.00000 |
|  | **RvD1** | 0.0001 | 0.0001 | 0.0001 | 0.0001 | 0.00000 |
|  | **PD1(n-3,DPA)** | 0.0001 | 0.0001 | 0.0001 | 0.0001 | 0.00000 |
|  | **PD1** | 0.0001 | 0.0001 | 0.0001 | 0.0001 | 0.00000 |
|  | **Maresin1** | 0.0001 | 0.0001 | 0.01056 | 0.00354 | 0.00608 |
|  | **MaR1(n-3,DPA)** | 0.02963 | 0.01710 | 0.02069 | 0.02247 | 0.00645 |
|  | **AT-RvD3** | 0.0001 | 0.0001 | 0.0001 | 0.0001 | 0.00000 |
|  | **AT-RvD1** | 0.0001 | 0.0001 | 0.0001 | 0.0001 | 0.00000 |
|  | **AT-PD1** | 0.0001 | 0.0001 | 0.0001 | 0.0001 | 0.00000 |
|  | **7(S)-Maresin1** | 0.0001 | 0.0001 | 0.0001 | 0.0001 | 0.00000 |
|  | **7-HDoHE** | 0.22119 | 0.23106 | 0.19085 | 0.21437 | 0.02096 |
|  | **4-HDoHE** | 0.69727 | 0.80056 | 0.67511 | 0.72431 | 0.06695 |
|  | **22-OH-PD1** | 0.0001 | 0.0001 | 0.0001 | 0.0001 | 0.00000 |
|  | **17-HDoHE** | 0.27734 | 0.25276 | 0.23783 | 0.25597 | 0.01995 |
|  | **14-HDoHE** | 1.11636 | 1.03229 | 1.07248 | 1.07371 | 0.04205 |
|  | **13-HDoHE** | 0.34132 | 0.34774 | 0.34041 | 0.34316 | 0.00399 |
|  | **10S,17S-DiHDoHE** | 0.0001 | 0.0001 | 0.0001 | 0.0001 | 0.00000 |

Supplemental Table 2: Concentration of lipid mediators in non-centrifuged CSF. Bolded values indicate the use of imputed values for more than 50% of samples, based on the lower limit of detection of 0.0001 ng/mL.

|  |  | **Pre-op (ng/ml)** | | | | **24 Hours Post-op (ng/ml)** | | | | **6 Weeks Post-op (ng/ml)** | | | |
| --- | --- | --- | --- | --- | --- | --- | --- | --- | --- | --- | --- | --- | --- |
| **Metabolome** | **Lipid Mediator** | **Median** | **Q1** | **Q3** | **IQR** | **Median** | **Q1** | **Q3** | **IQR** | **Median** | **Q1** | **Q3** | **IQR** |
| **AA** | **TXB2** | 0.0238 | 0.0151 | 0.0385 | 0.0234 | 0.0196 | 0.0106 | 0.0288 | 0.0182 | 0.0159 | 0.0089 | 0.0273 | 0.0184 |
|  | **PGF2a** | **0.0001** | **0.0001** | **0.0058** | **0.0057** | **0.0001** | **0.0001** | **0.0154** | **0.0153** | **0.0001** | **0.0001** | **0.0073** | **0.0072** |
|  | **PGE2** | 0.0081 | 0.0052 | 0.0134 | 0.0082 | 0.0128 | 0.0047 | 0.0232 | 0.0185 | 0.0075 | 0.0052 | 0.0103 | 0.0051 |
|  | **PGD2** | **0.0001** | **0.0001** | **0.0001** | **0** | **0.0001** | **0.0001** | **0.0001** | **0** | **0.0001** | **0.0001** | **0.0001** | **0** |
|  | **LXB4** | **0.0001** | **0.0001** | **0.0001** | **0** | **0.0001** | **0.0001** | **0.0001** | **0** | **0.0001** | **0.0001** | **0.0001** | **0** |
|  | **LXA5** | **0.0001** | **0.0001** | **0.0001** | **0** | **0.0001** | **0.0001** | **0.0006** | **0.0005** | **0.0001** | **0.0001** | **0.0001** | **0** |
|  | **LXA4** | **0.0001** | **0.0001** | **0.0001** | **0** | **0.0001** | **0.0001** | **0.0001** | **0** | **0.0001** | **0.0001** | **0.0001** | **0** |
|  | **LTB4** | **0.0001** | **0.0001** | **0.0001** | **0** | **0.0001** | **0.0001** | **0.0001** | **0** | **0.0001** | **0.0001** | **0.0001** | **0** |
|  | **5(S),6(R)-DiHETE** | **0.0001** | **0.0001** | **0.0001** | **0** | **0.0001** | **0.0001** | **0.0001** | **0** | **0.0001** | **0.0001** | **0.0001** | **0** |
|  | **5(S),15(S)-DiHETE** | **0.0001** | **0.0001** | **0.0001** | **0** | **0.0001** | **0.0001** | **0.0001** | **0** | **0.0001** | **0.0001** | **0.0001** | **0** |
|  | **5(S),12(S)-DiHETE** | **0.0002** | **0.0001** | **0.0012** | **0.0011** | **0.0001** | **0.0001** | **0.0008** | **0.0007** | 0.0005 | 0.0001 | 0.0012 | 0.0011 |
|  | **5-HETE** | 0.0020 | 0.0014 | 0.0029 | 0.0015 | 0.0019 | 0.0014 | 0.003 | 0.0016 | 0.0018 | 0.0013 | 0.0025 | 0.0012 |
|  | **20-hydroxy LTB4** | **0.0001** | **0.0001** | **0.0001** | **0** | **0.0001** | **0.0001** | **0.0001** | **0** | **0.0001** | **0.0001** | **0.0001** | **0** |
|  | **20-COOH LTB4** | **0.0001** | **0.0001** | **0.0001** | **0** | **0.0001** | **0.0001** | **0.0001** | **0** | **0.0001** | **0.0001** | **0.0001** | **0** |
|  | **15-HETE** | 0.0016 | 0.0012 | 0.0025 | 0.0014 | 0.0013 | 0.0009 | 0.0019 | 0.001 | 0.0014 | 0.001 | 0.002 | 0.001 |
|  | **15-epi LXA4** | **0.0001** | **0.0001** | **0.0001** | **0** | **0.0001** | **0.0001** | **0.0001** | **0** | **0.0001** | **0.0001** | **0.0001** | **0** |
|  | **12(S)-HHTrE** | **0.0001** | **0.0001** | **0.0004** | **0.0003** | **0.0001** | **0.0001** | **0.0006** | **0.0005** | **0.0001** | **0.0001** | **0.0001** | **0** |
|  | **12-HETE** | 0.0041 | 0.0029 | 0.0066 | 0.0037 | 0.0034 | 0.0022 | 0.0053 | 0.0031 | 0.004 | 0.0027 | 0.0071 | 0.0043 |
|  | **11-HETE** | 0.0030 | 0.0024 | 0.0044 | 0.002 | 0.0022 | 0.0016 | 0.0035 | 0.0019 | 0.0029 | 0.0021 | 0.0038 | 0.0017 |
| **DHA** | **RvD6** | **0.0001** | **0.0001** | **0.0001** | **0** | **0.0001** | **0.0001** | **0.0001** | **0** | **0.0001** | **0.0001** | **0.0001** | **0** |
|  | **RvD5(n-3,DPA)** | **0.0001** | **0.0001** | **0.0001** | **0** | **0.0001** | **0.0001** | **0.0001** | **0** | **0.0001** | **0.0001** | **0.0001** | **0** |
|  | **RvD5** | **0.0001** | **0.0001** | **0.0001** | **0** | **0.0001** | **0.0001** | **0.0001** | **0** | **0.0001** | **0.0001** | **0.0001** | **0** |
|  | **RvD4** | **0.0001** | **0.0001** | **0.0001** | **0** | **0.0001** | **0.0001** | **0.0001** | **0** | **0.0001** | **0.0001** | **0.0001** | **0** |
|  | **RvD3** | **0.0001** | **0.0001** | **0.0001** | **0** | **0.0001** | **0.0001** | **0.0001** | **0** | **0.0001** | **0.0001** | **0.0001** | **0** |
|  | **RvD2** | **0.0001** | **0.0001** | **0.0001** | **0** | **0.0001** | **0.0001** | **0.0001** | **0** | **0.0001** | **0.0001** | **0.0001** | **0** |
|  | **RvD1** | **0.0001** | **0.0001** | **0.0001** | **0** | **0.0001** | **0.0001** | **0.0001** | **0** | **0.0001** | **0.0001** | **0.0001** | **0** |
|  | **PD1(n-3,DPA)** | 0.0648 | 0.0001 | 0.1063 | 0.1062 | 0.0467 | 0.0001 | 0.0966 | 0.0965 | 0.038 | 0.0001 | 0.0882 | 0.0881 |
|  | **PD1** | **0.0001** | **0.0001** | **0.0001** | **0** | **0.0001** | **0.0001** | **0.0001** | **0** | **0.0001** | **0.0001** | **0.0001** | **0** |
|  | **Maresin1** | **0.0001** | **0.0001** | **0.0001** | **0** | **0.0001** | **0.0001** | **0.0001** | **0** | **0.0001** | **0.0001** | **0.0001** | **0** |
|  | **MaR1(n-3,DPA)** | **0.0001** | **0.0001** | **0.0001** | **0** | **0.0001** | **0.0001** | **0.0001** | **0** | **0.0001** | **0.0001** | **0.0001** | **0** |
|  | **AT-RvD3** | **0.0001** | **0.0001** | **0.0001** | **0** | **0.0001** | **0.0001** | **0.0001** | **0** | **0.0001** | **0.0001** | **0.0001** | **0** |
|  | **AT-RvD1** | **0.0001** | **0.0001** | **0.0001** | **0** | **0.0001** | **0.0001** | **0.0001** | **0** | **0.0001** | **0.0001** | **0.0001** | **0** |
|  | **AT-PD1** | **0.0001** | **0.0001** | **0.0001** | **0** | **0.0001** | **0.0001** | **0.0001** | **0** | **0.0001** | **0.0001** | **0.0001** | **0** |
|  | **7(S)-Maresin1** | **0.0001** | **0.0001** | **0.0001** | **0** | **0.0001** | **0.0001** | **0.0001** | **0** | **0.0001** | **0.0001** | **0.0001** | **0** |
|  | **7-HDoHE** | **0.0001** | **0.0001** | **0.0001** | **0** | **0.0001** | **0.0001** | **0.0001** | **0** | **0.0001** | **0.0001** | **0.0001** | **0** |
|  | **4-HDoHE** | 0.0011 | 0.0009 | 0.0017 | 0.0008 | 0.0011 | 0.0008 | 0.0016 | 0.0008 | 0.0011 | 0.0009 | 0.0014 | 0.0006 |
|  | **22-OH-PD1** | **0.0001** | **0.0001** | **0.0001** | **0** | **0.0001** | **0.0001** | **0.0001** | **0** | **0.0001** | **0.0001** | **0.0001** | **0** |
|  | **17-HDoHE** | **0.0001** | **0.0001** | **0.0001** | **0** | **0.0001** | **0.0001** | **0.0001** | **0** | **0.0001** | **0.0001** | **0.0001** | **0** |
|  | **14-HDoHE** | 0.0005 | 0.0003 | 0.0008 | 0.0005 | 0.0004 | 0.0002 | 0.0006 | 0.0004 | 0.0005 | 0.0003 | 0.0007 | 0.0003 |
|  | **13-HDoHE** | 0.0007 | 0.0005 | 0.0009 | 0.0004 | 0.0005 | 0.0003 | 0.0007 | 0.0003 | 0.0006 | 0.0005 | 0.0008 | 0.0003 |
|  | **10S,17S-DiHDoHE** | **0.0001** | **0.0001** | **0.0018** | **0.0017** | **0.0001** | **0.0001** | **0.0025** | **0.0024** | **0.0001** | **0.0001** | **0.0034** | **0.0033** |
| **EPA** | **RvE3** | 0.0033 | 0.0019 | 0.0045 | 0.0026 | 0.0029 | 0.002 | 0.0043 | 0.0024 | 0.0033 | 0.0025 | 0.0048 | 0.0024 |
|  | **RvE2** | **0.0001** | **0.0001** | **0.0001** | **0** | **0.0001** | **0.0001** | **0.0001** | **0** | **0.0001** | **0.0001** | **0.0001** | **0** |
|  | **RvE1** | **0.0001** | **0.0001** | **0.0001** | **0** | **0.0001** | **0.0001** | **0.0001** | **0** | **0.0001** | **0.0001** | **0.0001** | **0** |
|  | **5-HEPE** | **0.0001** | **0.0001** | **0.0001** | **0** | **0.0001** | **0.0001** | **0.0001** | **0** | **0.0001** | **0.0001** | **0.0001** | **0** |
|  | **18-HEPE** | 0.0003 | 0.0001 | 0.0006 | 0.0005 | **0.0001** | **0.0001** | **0.0004** | **0.0003** | 0.0003 | 0.0001 | 0.0005 | 0.0004 |
|  | **15(S)-HEPE** | **0.0001** | **0.0001** | **0.0001** | **0** | **0.0001** | **0.0001** | **0.0001** | **0** | **0.0001** | **0.0001** | **0.0001** | **0** |
|  | **12-HEPE** | **0.0001** | **0.0001** | **0.0001** | **0** | **0.0001** | **0.0001** | **0.0001** | **0** | **0.0001** | **0.0001** | **0.0001** | **0** |
|  | **11-HEPE** | **0.0001** | **0.0001** | **0.0001** | **0** | **0.0001** | **0.0001** | **0.0001** | **0** | **0.0001** | **0.0001** | **0.0001** | **0** |
|  | **5(S),15(S)-DiHEPE** | **0.0001** | **0.0001** | **0.0047** | **0.0046** | **0.0001** | **0.0001** | **0.0036** | **0.0035** | 0.0024 | 0.0001 | 0.005 | 0.0049 |

Supplemental Table 3: Concentration of lipid mediators in centrifuged CSF cell pellets after subtracting FBS + 10% DMSO baseline levels. For analytes that had a lower median value in CSF cell pellets than vehicle (FBS with 10% DMSO; see supplemental table 1), we imputed a concentration value of 0. Bolded values indicate the use of imputed values for more than 50% of samples, based on the lower limit of detection of 0.0001 ng/mL. Pellets were obtained from centrifugation of ~12 mL of CSF. Quartile and interquartile ranges below 0 are listed as “N/A.”

|  |  | **Pre-op (ng/ml)** | | | | **24 Hours Post-op (ng/ml)** | | | | **6 Weeks Post-op (ng/ml)** | | | |
| --- | --- | --- | --- | --- | --- | --- | --- | --- | --- | --- | --- | --- | --- |
| **Metabolome** | **Lipid** | **Median** | **Q1** | **Q3** | **IQR** | **Median** | **Q1** | **Q3** | **IQR** | **Median** | **Q1** | **Q3** | **IQR** |
| **AA** | **TXB2** | 0.0000 | N/A | N/A | N/A | 0.0000 | N/A | N/A | N/A | 0.0000 | N/A | N/A | N/A |
|  | **PGF2a** | 0.0039 | 0.0001 | 0.0093 | 0.0092 | 0.0050 | 0.0014 | 0.0136 | 0.0122 | 0.0070 | 0.0035 | 0.0232 | 0.0197 |
|  | **PGE2** | 0.6430 | 0.0200 | 0.9425 | 0.9225 | 0.5187 | 0.0145 | 1.0449 | 1.0304 | 0.6517 | 0.0173 | 1.1015 | 1.0842 |
|  | **PGD2** | **0.0001** | **0.0001** | **0.0001** | **0.0000** | **0.0001** | **0.0001** | **0.0001** | **0.0000** | **0.0001** | **0.0001** | **0.0001** | **0.0000** |
|  | **LXB4** | **0.0001** | **0.0001** | **0.0001** | **0.0000** | **0.0001** | **0.0001** | **0.0001** | **0.0000** | **0.0001** | **0.0001** | **0.0001** | **0.0000** |
|  | **LXA5** | **0.0001** | **0.0001** | **0.0001** | **0.0000** | **0.0001** | **0.0001** | **0.0001** | **0.0000** | **0.0001** | **0.0001** | **0.0016** | **0.0015** |
|  | **LXA4** | 0.1428 | 0.0041 | 0.1783 | 0.1742 | 0.1334 | 0.0033 | 0.1896 | 0.1863 | 0.1480 | 0.0039 | 0.2342 | 0.2304 |
|  | **LTB4** | 0.0882 | 0.0001 | 0.1076 | 0.1075 | 0.0951 | 0.0001 | 0.1297 | 0.1296 | 0.1028 | 0.0001 | 0.1532 | 0.1531 |
|  | **5(S),6(R)-DiHETE** | 0.1020 | 0.0075 | 0.1579 | 0.1503 | 0.0122 | 0.0062 | 0.1301 | 0.1239 | 0.1322 | 0.0069 | 0.2406 | 0.2337 |
|  | **5(S),15(S)-DiHETE** | 0.0303 | 0.0020 | 0.0407 | 0.0387 | 0.0255 | 0.0018 | 0.0431 | 0.0412 | 0.0301 | 0.0022 | 0.0568 | 0.0545 |
|  | **5(S),12(S)-DiHETE** | 0.1665 | 0.0135 | 0.2199 | 0.2064 | 0.1431 | 0.0111 | 0.2287 | 0.2176 | 0.1656 | 0.0109 | 0.2662 | 0.2553 |
|  | **5-HETE** | 0.7327 | N/A | 1.1265 | N/A | 0.6938 | N/A | 1.1089 | N/A | 0.8911 | N/A | 1.4464 | N/A |
|  | **20-hydroxy LTB4** | **0.0001** | **0.0001** | **0.0001** | **0.0000** | **0.0001** | **0.0001** | **0.0001** | **0.0000** | **0.0001** | **0.0001** | **0.0001** | **0.0000** |
|  | **20-COOH LTB4** | **0.0001** | **0.0001** | **0.0001** | **0.0000** | **0.0001** | **0.0001** | **0.0001** | **0.0000** | **0.0001** | **0.0001** | **0.0001** | **0.0000** |
|  | **15-HETE** | 0.4536 | N/A | 0.6179 | N/A | 0.3841 | N/A | 0.6369 | N/A | 0.4163 | N/A | 0.7461 | N/A |
|  | **15-epi LXA4** | 0.0035 | 0.0001 | 0.0847 | 0.0846 | 0.0028 | 0.0013 | 0.0532 | 0.0519 | 0.0918 | 0.0030 | 0.1598 | 0.1568 |
|  | **12(S)-HHTrE** | 0.0000 | N/A | 0.0154 | N/A | 0.0000 | N/A | 0.0020 | N/A | 0.0276 | N/A | 0.0346 | N/A |
|  | **12-HETE** | 0.5310 | 0.1280 | 1.4201 | 1.2921 | 0.4645 | 0.0935 | 0.9239 | 0.8303 | 2.8872 | 0.4414 | 3.4902 | 3.0488 |
|  | **11-HETE** | 0.4233 | 0.0459 | 0.6297 | 0.5838 | 0.4550 | 0.0376 | 0.6413 | 0.6037 | 0.4736 | 0.0645 | 0.8265 | 0.7620 |
| **DHA** | **RvD6** | **0.0001** | **0.0001** | **0.0023** | **0.0022** | **0.0001** | **0.0001** | **0.0015** | **0.0014** | **0.0001** | **0.0001** | **0.0024** | **0.0023** |
|  | **RvD5(n-3,DPA)** | 0.0011 | 0.0001 | 0.0026 | 0.0025 | 0.0015 | 0.0001 | 0.0028 | 0.0027 | 0.0019 | 0.0001 | 0.0040 | 0.0039 |
|  | **RvD5** | **0.0001** | **0.0001** | **0.0001** | **0.0000** | **0.0001** | **0.0001** | **0.0001** | **0.0000** | **0.0001** | **0.0001** | **0.0001** | **0.0000** |
|  | **RvD4** | **0.0001** | **0.0001** | **0.0001** | **0.0000** | **0.0001** | **0.0001** | **0.0001** | **0.0000** | **0.0001** | **0.0001** | **0.0004** | **0.0003** |
|  | **RvD3** | **0.0001** | **0.0001** | **0.0026** | **0.0025** | **0.0001** | **0.0001** | **0.0001** | **0.0000** | **0.0001** | **0.0001** | **0.0001** | **0.0000** |
|  | **RvD2** | **0.0001** | **0.0001** | **0.0135** | **0.0134** | 0.0054 | 0.0001 | 0.0138 | 0.0137 | 0.0119 | 0.0001 | 0.0182 | 0.0181 |
|  | **RvD1** | **0.0001** | **0.0001** | **0.0001** | **0.0000** | **0.0001** | **0.0001** | **0.0001** | **0.0000** | **0.0001** | **0.0001** | **0.0004** | **0.0003** |
|  | **PD1(n-3,DPA)** | **0.0001** | **0.0001** | **0.0001** | **0.0000** | **0.0001** | **0.0001** | **0.0001** | **0.0000** | **0.0001** | **0.0001** | **0.0001** | **0.0000** |
|  | **PD1** | **0.0001** | **0.0001** | **0.0001** | **0.0000** | **0.0001** | **0.0001** | **0.0001** | **0.0000** | **0.0001** | **0.0001** | **0.0001** | **0.0000** |
|  | **Maresin1** | **0.0001** | **0.0001** | **0.0031** | **0.0030** | **0.0001** | **0.0001** | **0.0023** | **0.0022** | **0.0001** | **0.0001** | **0.0038** | **0.0037** |
|  | **MaR1(n-3,DPA)** | **0.0001** | **0.0001** | **0.0077** | **0.0076** | **0.0001** | **0.0001** | **0.0067** | **0.0066** | **0.0001** | **0.0001** | **0.0092** | **0.0091** |
|  | **AT-RvD3** | **0.0001** | **0.0001** | **0.0089** | **0.0088** | **0.0001** | **0.0001** | **0.0091** | **0.0090** | **0.0001** | **0.0001** | **0.0013** | **0.0012** |
|  | **AT-RvD1** | **0.0001** | **0.0001** | **0.0003** | **0.0002** | **0.0001** | **0.0001** | **0.0001** | **0.0000** | **0.0001** | **0.0001** | **0.0001** | **0.0000** |
|  | **AT-PD1** | **0.0001** | **0.0001** | **0.0001** | **0.0000** | **0.0001** | **0.0001** | **0.0001** | **0.0000** | **0.0001** | **0.0001** | **0.0001** | **0.0000** |
|  | **7(S)-Maresin1** | **0.0001** | **0.0001** | **0.0001** | **0.0000** | **0.0001** | **0.0001** | **0.0001** | **0.0000** | **0.0001** | **0.0001** | **0.0001** | **0.0000** |
|  | **7-HDoHE** | 0.0405 | 0.0005 | 0.0520 | 0.0515 | 0.0428 | N/A | 0.0518 | N/A | 0.0430 | 0.0016 | 0.0794 | 0.0777 |
|  | **4-HDoHE** | 0.1790 | 0.0445 | 0.2913 | 0.2469 | 0.2090 | 0.0315 | 0.3533 | 0.3218 | 0.2084 | 0.0395 | 0.3589 | 0.3194 |
|  | **22-OH-PD1** | **0.0001** | **0.0001** | **0.0004** | **0.0003** | 0.0002 | 0.0001 | 0.0004 | 0.0003 | 0.0003 | 0.0001 | 0.0005 | 0.0004 |
|  | **17-HDoHE** | 0.0468 | 0.0006 | 0.0610 | 0.0604 | 0.0435 | N/A | 0.0630 | N/A | 0.0469 | 0.0023 | 0.0818 | 0.0795 |
|  | **14-HDoHE** | 0.0575 | 0.0066 | 0.0943 | 0.0877 | 0.0583 | 0.0034 | 0.0963 | 0.0929 | 0.0683 | 0.0179 | 0.1280 | 0.1101 |
|  | **13-HDoHE** | 0.1138 | 0.0038 | 0.1564 | 0.1526 | 0.1116 | 0.0022 | 0.1614 | 0.1591 | 0.1194 | 0.0084 | 0.1981 | 0.1897 |
|  | **10S,17S-DiHDoHE** | 0.0790 | 0.0045 | 0.0941 | 0.0896 | 0.0700 | 0.0048 | 0.0995 | 0.0947 | 0.0770 | 0.0054 | 0.1237 | 0.1183 |
| **EPA** | **RvE3** | 0.0049 | 0.0001 | 0.0074 | 0.0073 | 0.0043 | 0.0001 | 0.0068 | 0.0067 | 0.0076 | 0.0001 | 0.0106 | 0.0105 |
|  | **RvE2** | 0.0008 | 0.0001 | 0.0017 | 0.0016 | 0.0005 | 0.0001 | 0.0017 | 0.0016 | 0.0014 | 0.0001 | 0.0023 | 0.0022 |
|  | **RvE1** | **0.0001** | **0.0001** | **0.0001** | **0.0000** | **0.0001** | **0.0001** | **0.0001** | **0.0000** | **0.0001** | **0.0001** | **0.0001** | **0.0000** |
|  | **5-HEPE** | 0.0189 | 0.0004 | 0.0235 | 0.0231 | 0.0158 | 0.0000 | 0.0252 | 0.0251 | 0.0178 | 0.0010 | 0.0307 | 0.0297 |
|  | **18-HEPE** | 0.0092 | 0.0012 | 0.0130 | 0.0118 | 0.0096 | 0.0011 | 0.0140 | 0.0129 | 0.0101 | 0.0016 | 0.0160 | 0.0144 |
|  | **15(S)-HEPE** | 0.0015 | N/A | 0.0040 | N/A | 0.0014 | N/A | 0.0038 | N/A | 0.0013 | 0.0006 | 0.0049 | 0.0042 |
|  | **12-HEPE** | 0.0000 | N/A | 0.0518 | N/A | 0.0003 | N/A | 0.0177 | N/A | 0.0645 | N/A | 0.0861 | N/A |
|  | **11-HEPE** | 0.0069 | 0.0026 | 0.0089 | 0.0063 | 0.0064 | 0.0023 | 0.0091 | 0.0068 | 0.0067 | 0.0033 | 0.0128 | 0.0094 |
|  | **5(S),15(S)-DiHEPE** | 0.0001 | 0.0001 | 0.0013 | 0.0012 | 0.0001 | 0.0001 | 0.0027 | 0.0026 | 0.0001 | 0.0001 | 0.0030 | 0.0029 |

Supplemental Table 4: Concentrations of lipid mediators in centrifuged CSF supernatants. Bolded values indicate the use of imputed values for more than 50% of samples, based on the lower limit of detection of 0.0001 ng/mL.

|  |  | **Pre-op (ng/ml)** | | | | **24 Hours Post-op (ng/ml)** | | | | **6 Weeks Post-op (ng/ml)** | | | |
| --- | --- | --- | --- | --- | --- | --- | --- | --- | --- | --- | --- | --- | --- |
| **Metabolome** | **Lipid** | **Median** | **Q1** | **Q3** | **IQR** | **Median** | **Q1** | **Q3** | **IQR** | **Median** | **Q1** | **Q3** | **IQR** |
| **AA** | **TXB2** | 0.0096 | 0.007 | 0.0172 | 0.0102 | 0.0087 | 0.005 | 0.0118 | 0.0069 | 0.0112 | 0.0072 | 0.0159 | 0.0087 |
|  | **PGF2a** | 0.0053 | 0.0045 | 0.0076 | 0.003 | 0.0049 | 0.0028 | 0.0068 | 0.004 | 0.0046 | 0.0025 | 0.007 | 0.0045 |
|  | **PGE2** | 0.0046 | 0.0035 | 0.0083 | 0.0049 | 0.0037 | 0.0031 | 0.0073 | 0.0041 | 0.0051 | 0.0034 | 0.0117 | 0.0083 |
|  | **PGD2** | **0.0001** | **0.0001** | **0.0029** | **0.0028** | **0.0001** | **0.0001** | **0.0001** | **0** | **0.0001** | **0.0001** | **0.0012** | **0.0011** |
|  | **LXB4** | **0.0001** | **0.0001** | **0.0001** | **0** | **0.0001** | **0.0001** | **0.0002** | **0.0001** | **0.0001** | **0.0001** | **0.0001** | **0** |
|  | **LXA5** | **0.0001** | **0.0001** | **0.0001** | **0** | **0.0001** | **0.0001** | **0.0001** | **0** | **0.0001** | **0.0001** | **0.0001** | **0** |
|  | **LXA4** | **0.0001** | **0.0001** | **0.0001** | **0** | **0.0001** | **0.0001** | **0.0001** | **0** | **0.0001** | **0.0001** | **0.0001** | **0** |
|  | **LTB4** | **0.0001** | **0.0001** | **0.0001** | **0** | **0.0001** | **0.0001** | **0.0001** | **0** | **0.0001** | **0.0001** | **0.0001** | **0** |
|  | **5(S),6(R)-DiHETE** | **0.0001** | **0.0001** | **0.0001** | **0** | **0.0001** | **0.0001** | **0.0001** | **0** | **0.0001** | **0.0001** | **0.0001** | **0** |
|  | **5(S),15(S)-DiHETE** | **0.0001** | **0.0001** | **0.0001** | **0** | **0.0001** | **0.0001** | **0.0001** | **0** | **0.0001** | **0.0001** | **0.0001** | **0** |
|  | **5(S),12(S)-DiHETE** | **0.0001** | **0.0001** | **0.0002** | **0.0001** | 0.0002 | 0.0001 | 0.0003 | 0.0002 | 0.0001 | 0.0001 | 0.0002 | 0.0001 |
|  | **5-HETE** | 0.0021 | 0.0014 | 0.0026 | 0.0011 | 0.0023 | 0.0014 | 0.0031 | 0.0017 | 0.0026 | 0.0019 | 0.0042 | 0.0024 |
|  | **20-hydroxy LTB4** | **0.0001** | **0.0001** | **0.0001** | **0** | **0.0001** | **0.0001** | **0.0001** | **0** | **0.0001** | **0.0001** | **0.0001** | **0** |
|  | **20-COOH LTB4** | **0.0001** | **0.0001** | **0.0001** | **0** | **0.0001** | **0.0001** | **0.0001** | **0** | **0.0001** | **0.0001** | **0.0001** | **0** |
|  | **15-HETE** | 0.0017 | 0.0014 | 0.0023 | 0.0009 | 0.0015 | 0.0013 | 0.0023 | 0.0011 | 0.002 | 0.0013 | 0.0029 | 0.0016 |
|  | **15-epi LXA4** | **0.0001** | **0.0001** | **0.0001** | **0** | **0.0001** | **0.0001** | **0.0001** | **0** | **0.0001** | **0.0001** | **0.0001** | **0** |
|  | **12(S)-HHTrE** | 0.0001 | 0.0001 | 0.0005 | 0.0004 | **0.0001** | **0.0001** | **0.0002** | **0.0001** | **0.0001** | **0.0001** | **0.0005** | **0.0004** |
|  | **12-HETE** | 0.0039 | 0.0025 | 0.0056 | 0.0031 | 0.0032 | 0.0021 | 0.0044 | 0.0023 | 0.0042 | 0.0027 | 0.0077 | 0.005 |
|  | **11-HETE** | 0.0037 | 0.0026 | 0.0049 | 0.0023 | 0.003 | 0.0024 | 0.0043 | 0.002 | 0.0036 | 0.0028 | 0.0057 | 0.0028 |
| **DHA** | **RvD6** | **0.0001** | **0.0001** | **0.0001** | **0** | **0.0001** | **0.0001** | **0.0001** | **0** | **0.0001** | **0.0001** | **0.0001** | **0** |
|  | **RvD5(n-3,DPA)** | **0.0001** | **0.0001** | **0.0001** | **0** | **0.0001** | **0.0001** | **0.0001** | **0** | **0.0001** | **0.0001** | **0.0001** | **0** |
|  | **RvD5** | **0.0001** | **0.0001** | **0.0001** | **0** | **0.0001** | **0.0001** | **0.0001** | **0** | **0.0001** | **0.0001** | **0.0001** | **0** |
|  | **RvD4** | **0.0001** | **0.0001** | **0.0001** | **0** | **0.0001** | **0.0001** | **0.0001** | **0** | **0.0001** | **0.0001** | **0.0001** | **0** |
|  | **RvD3** | **0.0001** | **0.0001** | **0.0001** | **0** | **0.0001** | **0.0001** | **0.0001** | **0** | **0.0001** | **0.0001** | **0.0001** | **0** |
|  | **RvD2** | **0.0001** | **0.0001** | **0.0001** | **0** | **0.0001** | **0.0001** | **0.0001** | **0** | **0.0001** | **0.0001** | **0.0001** | **0** |
|  | **RvD1** | **0.0001** | **0.0001** | **0.0001** | **0** | **0.0001** | **0.0001** | **0.001** | **0.0009** | **0.0001** | **0.0001** | **0.0017** | **0.0016** |
|  | **PD1(n-3,DPA)** | **0.0001** | **0.0001** | **0.0001** | **0** | **0.0001** | **0.0001** | **0.0001** | **0** | **0.0001** | **0.0001** | **0.0001** | **0** |
|  | **PD1** | 0.0043 | 0.0001 | 0.0124 | 0.0123 | 0.0043 | 0.0001 | 0.0177 | 0.0176 | 0.0055 | 0.0001 | 0.0118 | 0.0117 |
|  | **Maresin1** | **0.0001** | **0.0001** | **0.0001** | **0** | **0.0001** | **0.0001** | **0.0001** | **0** | **0.0001** | **0.0001** | **0.0001** | **0** |
|  | **MaR1(n-3,DPA)** | **0.0001** | **0.0001** | **0.0001** | **0** | **0.0001** | **0.0001** | **0.0001** | **0** | **0.0001** | **0.0001** | **0.0001** | **0** |
|  | **AT-RvD3** | **0.0001** | **0.0001** | **0.0001** | **0** | **0.0001** | **0.0001** | **0.0001** | **0** | **0.0001** | **0.0001** | **0.0001** | **0** |
|  | **AT-RvD1** | **0.0001** | **0.0001** | **0.0001** | **0** | **0.0001** | **0.0001** | **0.0001** | **0** | **0.0001** | **0.0001** | **0.0001** | **0** |
|  | **AT-PD1** | **0.0001** | **0.0001** | **0.0001** | **0** | **0.0001** | **0.0001** | **0.0001** | **0** | **0.0001** | **0.0001** | **0.0001** | **0** |
|  | **7(S)-Maresin1** | **0.0001** | **0.0001** | **0.0003** | **0.0002** | **0.0001** | **0.0001** | **0.0001** | **0** | **0.0001** | **0.0001** | **0.0001** | **0** |
|  | **7-HDoHE** | **0.0001** | **0.0001** | **0.0001** | **0** | **0.0001** | **0.0001** | **0.0001** | **0** | **0.0001** | **0.0001** | **0.0001** | **0** |
|  | **4-HDoHE** | 0.0011 | 0.0009 | 0.0013 | 0.0004 | 0.001 | 0.0006 | 0.0014 | 0.0008 | 0.001 | 0.0006 | 0.0013 | 0.0007 |
|  | **22-OH-PD1** | **0.0001** | **0.0001** | **0.0001** | **0** | **0.0001** | **0.0001** | **0.0001** | **0** | **0.0001** | **0.0001** | **0.0001** | **0** |
|  | **17-HDoHE** | 0.0003 | 0.0001 | 0.0006 | 0.0005 | **0.0001** | **0.0001** | **0.0003** | **0.0002** | 0.0003 | 0.0001 | 0.0008 | 0.0007 |
|  | **14-HDoHE** | 0.0005 | 0.0003 | 0.0006 | 0.0002 | 0.0004 | 0.0002 | 0.0007 | 0.0005 | 0.0004 | 0.0001 | 0.0008 | 0.0007 |
|  | **13-HDoHE** | 0.0007 | 0.0006 | 0.001 | 0.0004 | 0.0005 | 0.0003 | 0.0008 | 0.0005 | 0.0007 | 0.0004 | 0.0012 | 0.0008 |
|  | **10S,17S-DiHDoHE** | **0.0001** | **0.0001** | **0.0001** | **0** | **0.0001** | **0.0001** | **0.0001** | **0** | **0.0001** | **0.0001** | **0.0006** | **0.0005** |
| **EPA** | **RvE3** | **0.0001** | **0.0001** | **0.0001** | **0** | **0.0001** | **0.0001** | **0.0001** | **0** | **0.0001** | **0.0001** | **0.0001** | **0** |
|  | **RvE2** | **0.0001** | **0.0001** | **0.0001** | **0** | **0.0001** | **0.0001** | **0.0001** | **0** | **0.0001** | **0.0001** | **0.0001** | **0** |
|  | **RvE1** | **0.0001** | **0.0001** | **0.0001** | **0** | **0.0001** | **0.0001** | **0.0001** | **0** | **0.0001** | **0.0001** | **0.0001** | **0** |
|  | **5-HEPE** | **0.0001** | **0.0001** | **0.0001** | **0** | **0.0001** | **0.0001** | **0.0001** | **0** | **0.0001** | **0.0001** | **0.0001** | **0** |
|  | **18-HEPE** | 0.0003 | 0.0003 | 0.0005 | 0.0002 | 0.0003 | 0.0001 | 0.0005 | 0.0004 | 0.0004 | 0.0002 | 0.0006 | 0.0004 |
|  | **15(S)-HEPE** | **0.0001** | **0.0001** | **0.0001** | **0** | **0.0001** | **0.0001** | **0.0001** | **0** | **0.0001** | **0.0001** | **0.0001** | **0** |
|  | **12-HEPE** | **0.0001** | **0.0001** | **0.0001** | **0** | **0.0001** | **0.0001** | **0.0001** | **0** | **0.0001** | **0.0001** | **0.0001** | **0** |
|  | **11-HEPE** | **0.0001** | **0.0001** | **0.0001** | **0** | **0.0001** | **0.0001** | **0.0001** | **0** | **0.0001** | **0.0001** | **0.0001** | **0** |
|  | **5(S),15(S)-DiHEPE** | **0.0001** | **0.0001** | **0.0001** | **0** | **0.0001** | **0.0001** | **0.0001** | **0** | **0.0001** | **0.0001** | **0.0001** | **0** |

Supplemental Figure 1: Non-centrifuged CSF heatmaps for AA, DHA, and EPA metabolomes from pre-op to 24 hours post-op, and 24 hours post-op to 6 weeks post-op. Patients are listed as numbers on the x-axis for each heatmap. All patients are presented, however only every other patient is listed due to size constraints. Heatmap scale numbers represent natural log ratios of analyte concentrations from pre-op to 24 hours post-op, and 24 hours post-op to 6 weeks post-op.


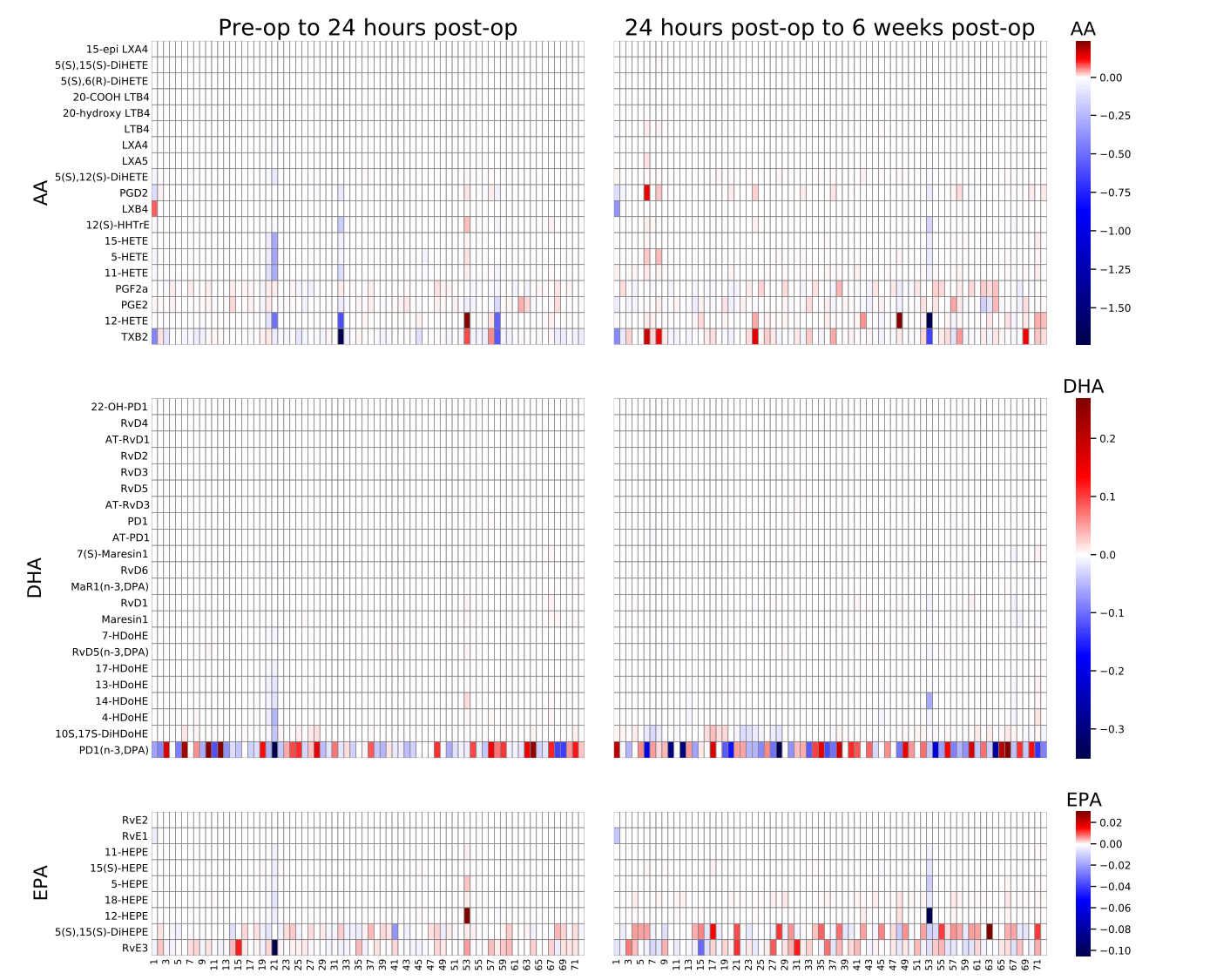


Supplemental Figure 2: Centrifuged CSF cell pellet heatmaps for AA, DHA, and EPA metabolomes from pre-op to 24 hours post-op, and 24 hours post-op to 6 weeks post-op. Patients are listed as numbers on the x-axis for each heatmap. Heatmap scale numbers represent natural log ratios of analyte concentrations from pre-op to 24 hours post-op, and 24 hours post-op to 6 weeks post-op.


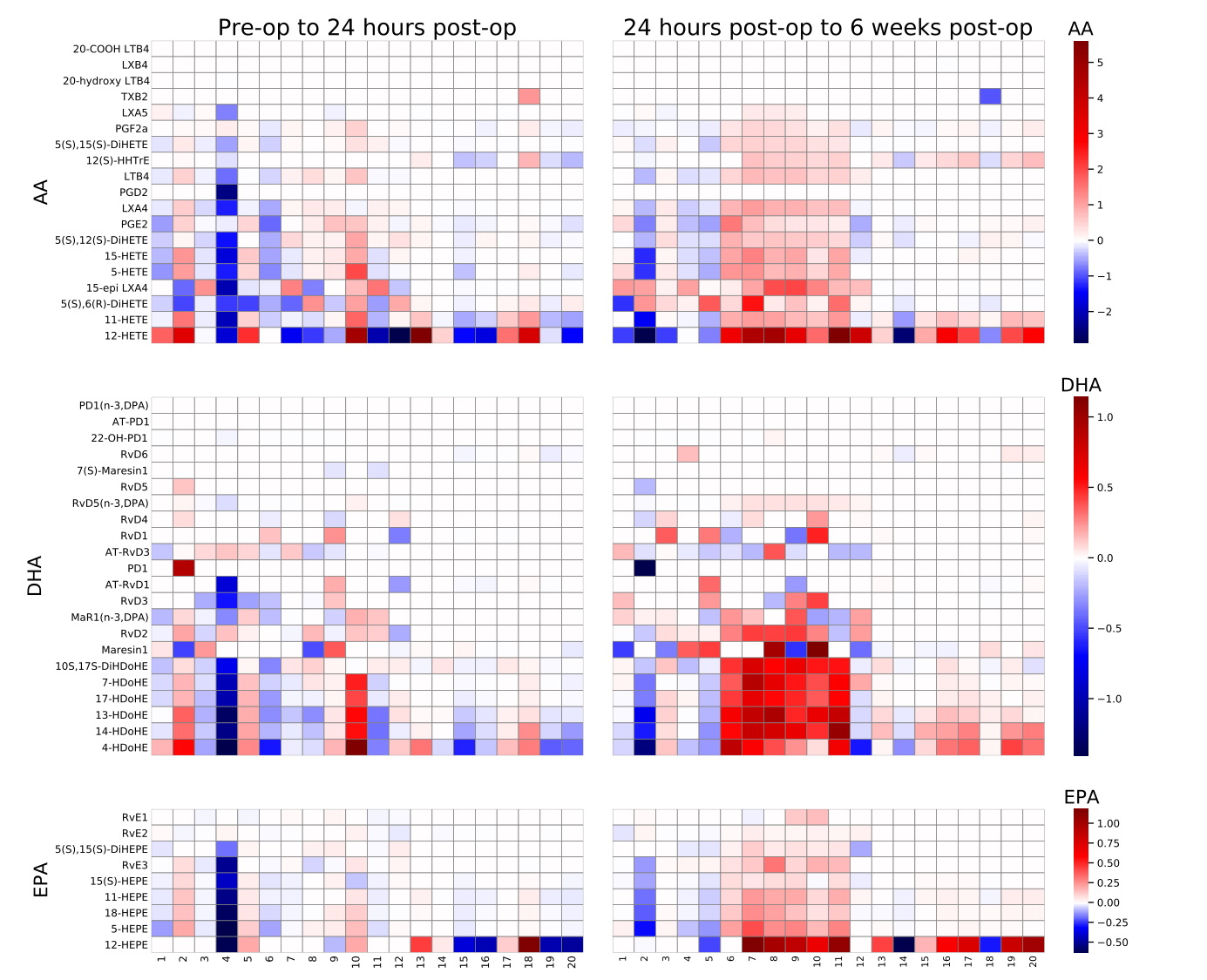


Supplemental Figure 3: Centrifuged CSF supernatant heatmaps for AA, DHA, and EPA metabolomes from pre-op to 24 hours post-op, and 24 hours post-op to 6 weeks post-op. Patients are listed as numbers on the x-axis for each heatmap. Heatmap scale numbers represent natural log ratios of analyte concentrations from pre-op to 24 hours post-op, and 24 hours post-op to 6 weeks post-op.


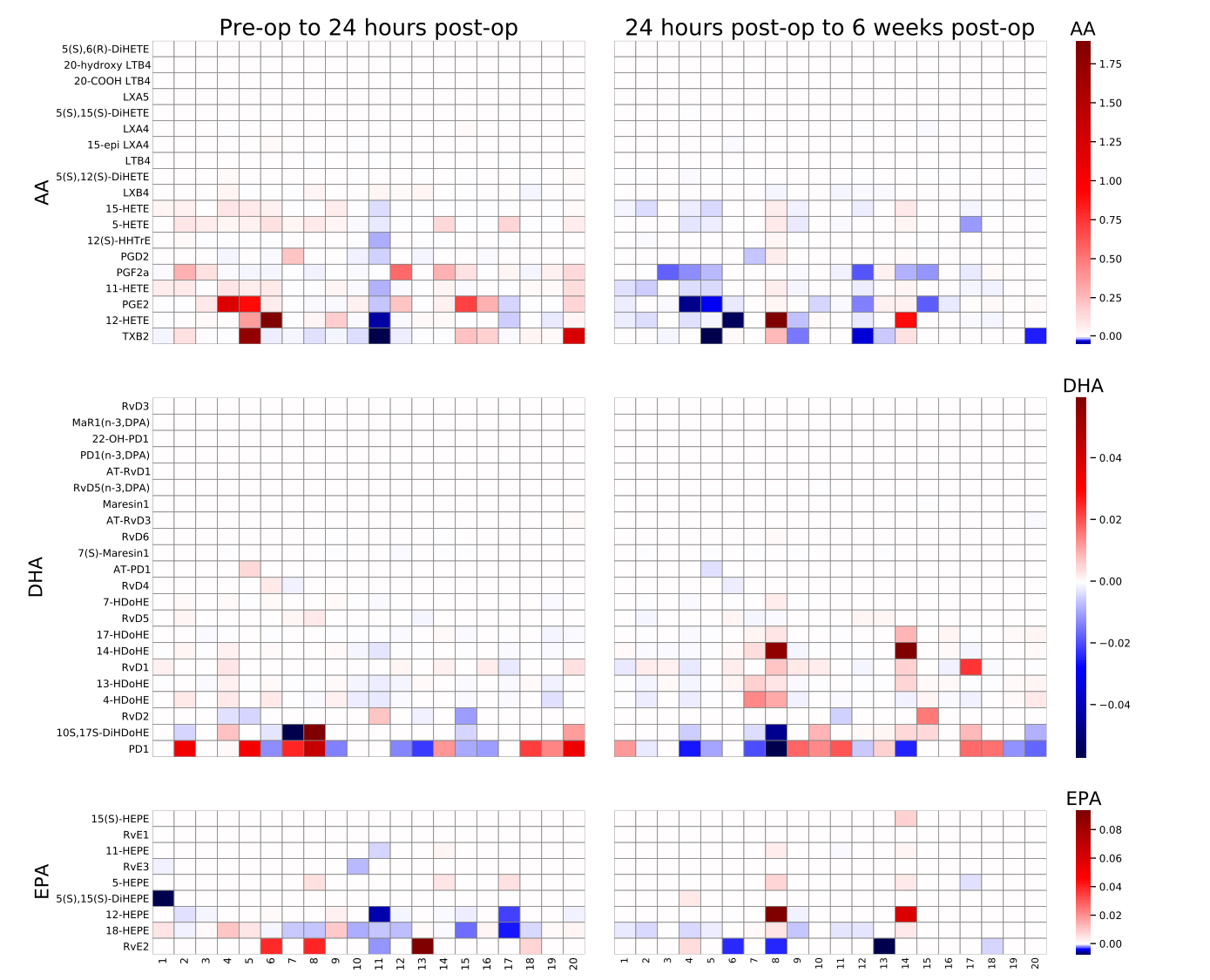

Supplement: Supplementary file 1 — Supplementary Information 1. [file 41598_2021_82606_MOESM1_ESM.docx]
